# Comparative mitochondrial genome analysis of three leafhopper species of the genus *Abrus* Dai & Zhang (Hemiptera: Cicadellidae: Deltocephalinae) from China with phylogenetic implication

**DOI:** 10.1186/s12864-023-09809-0

**Published:** 2023-11-27

**Authors:** Muhammad Asghar Hassan, Zhixiang Tan, Rongrong Shen, Jichun Xing

**Affiliations:** 1 Institute of Entomology, The Provincial Special Key Laboratory for Development and Utilization of Insect Resources, Guizhou University, Guiyang, 550025 Guizhou China; 2 Department of Biological Sciences, University of Memphis, Memphis, TN 38152 USA

**Keywords:** Leafhopper, Nucleotide composition, Genetic diversity, Mitogenome, Phylogenetic relationship

## Abstract

**Background:**

The phylogenetic position and classification of Athysanini are poorly defined, as it includes a large group of polyphyletic genera that have historically been assigned to it mainly because they still exhibit the most typical deltocephaline genitalic and external body characters but lack the distinctive characteristics that other tribes possess. The bamboo-feeding leafhopper genus *Abrus* belong to the tribe Athysanini of subfamily Deltocephalinae, which currently comprises 19 valid described species, and are limited to the Oriental and Palaearctic regions in China. Although the taxonomy of *Abrus* are well updated, the references on comparative mitogenomic analyses of *Abrus* species are only known for a single species. In this study, we sequenced and analyzed the complete mitochondrial genomes (mitogenomes) of *Abrus daozhenensis* Chen, Yang & Li, 2012 (16,391bp) and *A. yunshanensis* Chen, Yang & Li, 2012 (15,768bp) (Athysanini), and compared with published mitogenome sequence of *A. expansivus* Xing & Li, 2014 (15,904bp).

**Results:**

These *Abrus* species shared highly conserved mitogenomes with similar gene order to that of the putative ancestral insect with 37 typical genes and a non-coding A + T-rich region. The nucleotide composition of these genomes is highly biased toward A + T nucleotides (76.2%, 76.3%, and 74.7%), AT-skews (0.091 to 0.095, and 0.095), negative GC-skews (− 0.138, − 0.161, and − 0.138), and codon usage. All 22 tRNA genes had typical cloverleaf secondary structures, except for *trnS1* (AGN) which lacks the dihydrouridine arm, and distinctively *trnG* in the mitogenome of *A. expansivus* lacks the TψC arm. Phylogenetic analyses based on 13 PCGs, 2 rRNA genes, and 22 tRNA genes consistently recovered the monophyletic Opsiini, Penthimiini, Selenocephalini, Scaphoideini, and Athysanini (except *Watanabella graminea*, previously sequenced species as *Chlorotettix nigromaculatus*) based on limited available mitogenome sequence data of 37 species.

**Conclusion:**

At present, *Abrus* belongs to the tribe Athysanini based on both morphological and molecular datasets, which is strongly supported in present phylogenetic analyses in both BI and ML methods using the six concatenated datasets: amino acid sequences and nucleotides from different combinations of protein-coding genes (PCGs), ribosomal RNA (rRNAs), and transfer RNA (tRNAs). Phylogenetic trees reconstructed herein based on the BI and ML analyses consistently recovered monophylitic Athysanini, except *Watanabella graminea* (Athysanini) in Opsiini with high support values.

**Supplementary Information:**

The online version contains supplementary material available at 10.1186/s12864-023-09809-0.

## Background

Insect mitochondrial genome is a small double-stranded circular molecule with remarkable conservation in size ranging from 14–20 kb that encodes 37 genes: 13 protein-coding genes, two ribosomal RNAs, 22 transfer RNAs genes, and a non-coding A + T-rich region (or control region) [1–3]. The representative mitochondrial (mt) genomes of almost all insect orders from higher-level to lower taxonomic ranks have extensively been utilized for studying phylogeny, population genetics, comparative and evolutionary genomics, molecular evolution, and identification at various taxonomic levels [3–5]. Due to their high genome copy numbers, multiple genome-level characteristics, relatively high evolutionary rate, and greater phylogenetic informativeness than single mitochondrial genes, mitogenome sequences have been widely used in various phylogenomic studies [3, 5, 6].

The leafhopper genus *Abrus* belonging to the tribe Athysanini of subfamily Deltocephalinae, was originally described by Dai & Zhang [7] with six new species (type species: *A. hengshanensis* Dai & Zhang, 2002) from China. After that, Li & Wang [8], Dai & Zhang [9], Li et al. [10], Chen et al. [11], Yang & Chen [12], and Xing & Li [13] further added 13 new species from the Oriental and Palaearctic parts of China. So far, *Abrus* is only restricted to China with 19 valid described species, which are quite similar in body coloration and difficult to distinguish based on external morphology, but the male genitalia with a unique structure of aedeagus are considerably differences among these species [13]. Among these, 19 species are widely distributed in the Oriental Region (Guizhou, Sichuan, Hunan, Hubei, Guangxi, Guangdong, Fujian, and Zhejiang), and only *A. coneus* is also distributed in the Palaearctic Region (China: Gansu) [13]. It belongs to the tribe Athysanini, which is one of the largest and most diverse tribes of the subfamily Deltocephalinae, which includes 228 genera and 1181 described species worldwide, with a majority of species serve as a vector of economically important plant diseases [14, 15]. The generic and species composition in Athysanini is not constant at present and is continuously changed with time due to ongoing taxonomic and systematic revisions, which include the discovery of new genera or transfer of described genera to other tribes [14–17]. At present, there are approximately 20 genera and 70 species of Athysanini in China [10, 13, 16, 17].

The phylogenetic position and classification of Athysanini are poorly defined, as it includes a large group of polyphyletic genera that have historically been assigned to it mainly because they still exhibit the most typical deltocephaline genitalic and external body characters but lack the distinctive characteristics that other tribes possess [14, 15]. Numerous phylogenetic studies consistently supported for a paraphyletic Athysanini with a large group of polyphyletic genera based on morphology alone [18] or various combinations of morphological and molecular datasets: two nuclear gene fragments (28S ribosomal DNA and Histone H3) [14, 19, 20], or different combinations of 13-37 mitochondrial genes [21, 22]. Despite being the largest tribe with a cosmopolitan distribution, so far, only the representative three genera (*Abrus*, *Norvellina*, and *Watanabella*) and five species of Athysanini have been sequenced and analyzed (including two novel sequences in this study) from China [23, 24].

In this study, we sequenced and analyzed the complete mitogenomes of *Abrus daozhenensis* and *Abrus yunshanensis*, and compared them with the published sequence of *Abrus expansivus* to examine the phylogenetic relationships among the newly sequenced and published athysanine species based on mtDNA genome data, using the concatenated amino acid sequences and nucleotide sequences from different combinations of protein-coding genes (PCGs), ribosomal RNA (rRNAs), and transfer RNA (tRNAs).

## Results

### General features and gene orders in *Abrus* mitogenomes

**Fig. 1:**
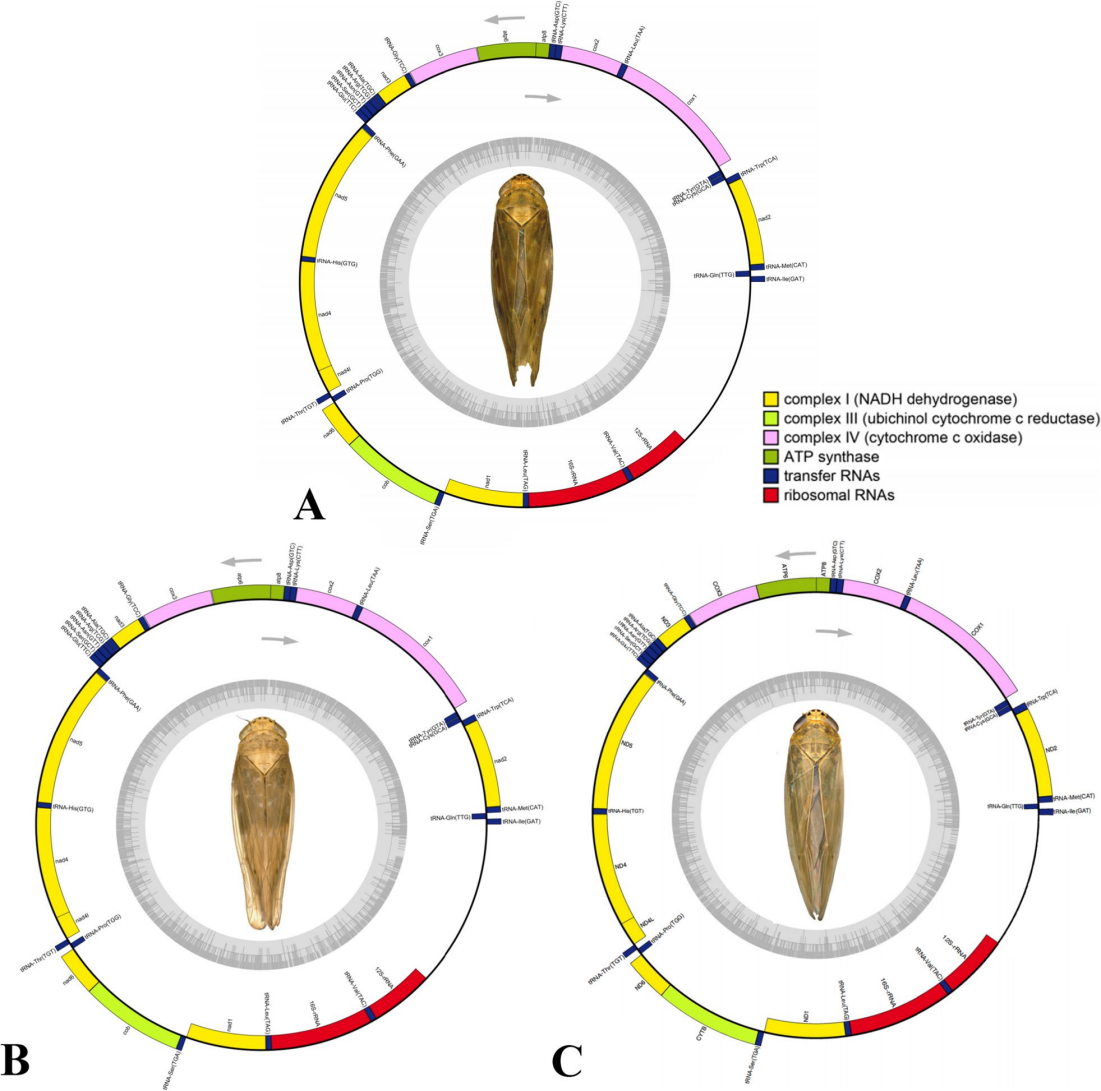
The complete mitochondrial genome maps of three *Abrus* species. **A** *Abrus daozhenensis*; **B** *Abrus yunshanensis*; **C** *Abrus expansivus*

The complete mitochondrial genomes of *A. daozhenensis* (GenBank: MZ274046), *A. yunshanensis* (GenBank: MZ274047), and *A. expansivus* (GenBank: MK033020) are 16,391 bp, 15,768 bp, and 15,904 bp in size, respectively (Table 1). The circular genome maps of these species are presented in Fig. 1A–C. All mitogenomes contained a typical set of 37 mitochondrial genes (13 PCGs, 22 tRNA genes, and two rRNA genes) and one control region (Supplementary Tables S1–S3). Gene order was invariant and identical to *Drosophila yakuba* and to other published deltocephaline mitogenomes [23, 25, 26].

**Table 1:** List of the mitochondrial genomes analyzed in the present study. List of taxa included in this study

**S. No.**	**Family**	**Subfamily**	**Tribe**	**Genus**	**Species**	**Accession No.**	**Length (bp)**	**References**
1	**CICADELLIDAE**	**Deltocephalinae**	**Penthimiini**	*Reticuluma*	*Reticuluma hamata*	MN922303	15,190	[27]
2				*Penthimia*	*Penthimia melanocephala*	MT768010	15,308	[27]
3			**Deltocephalini**	*Alobaldia*	*Alobaldia tobae*	KY039116	16,026	[28]
4				*Maiestas*	*Maiestas dorsalis*	NC036296	15,352	[29]
5			**Macrostelini**	*Cicadulina*	*Cicadulina mbila*	MK251127	12,554	[28]
6				*Balclutha*	*Balclutha* sp.	KX437738	14,819	[30]
7				*Macrosteles*	*Macrosteles quadrilineatus*	KY645960	16,626	[31]
8					*Macrosteles quadrimaculatus*	NC039560	15,734	[32]
9			**Mukariini**	*Mukaria*	*Mukaria splendida*	MG814385	16,711	[33]
10			**Chiasmini**	*Exitianus*	*Exitianus indicus*	KY039128	16,089	[30]
11				*Nephotettix*	*Nephotettix cincticeps*	KP749836	14,805	DS
12				*Aconurella*	*Aconurella prolixa*	MZ433366	14,832	[22]
13			**Paralimnini**	*Paralaevicephalus*	*Paralaevicephalus gracilipenis*	MK450366	16,114	[34]
14				*Psammotettix*	*Psammotettix* sp.	KX437742	12,970	[30]
15				*Yanocephalus*	*Yanocephalus yanonis*	NC036131	15,623	[28]
16			**Athysanini**	*Abrus*	*Abrus expansivus*	NC045238	15,904	[23]
17					*Abrus daozhenensis*	MZ274046	16,391	**Present study**
18					*Abrus yunshanensis*	MZ274047	15,768	**Present study**
19				*Norvellina*	*Norvellina* sp.	KY039131	15,594	[28]
20				*Watanabell*	*Watanabella graminea* (=*Chlorotettix nigromaculatus*)	MK234840	15,011	[24]
21			**Selenocephalini**	*Tambocerus*	*Tambocerus* sp.	KT827824	15,955	[35]
22			**Opsiini**	*Orosius*	*Orosius orientalis*	KY039146	15,513	[28]
23				*Hishimonoides*	*Hishimonoides recurvatis*	KY364883	14,184	DS
24				*Hishimonus*	*Hishimonus phycitis*	KX437727	11,328	[30]
25				*Japananus*	*Japananus hyalinus*	NC036298	15,364	[29]
26			**Scaphoideini**	*Scaphoideus*	*Scaphoideus maai*	KY817243	15,188	[26]
27					*Scaphoideus nigrivalveus*	KY817244	15,235	[26]
28					*Scaphoideus varius*	KY817245	15,207	[26]
20				*Phlogotettix*	*Phlogotettix* sp.	KY039135	15,136	[28]
30			**Eupelicini**	*Paradorydium*	*Paradorydium reflexana*	MG813487	15,661	DS
31			**Drabescini**	*Drabescoides*	*Drabescoides nuchalis*	NC028154	15,309	[36]
32				*Drabescus*	*Drabescus ineffectus*	MT527188	15,744	[37]
33				*Dryadomorpha*	*Dryadomorpha* sp.	KX437736	12,297	[30]
34				*Athysanopsis*	*Athysanopsis* sp.	KX437726	14,753	[28]
35				*Roxasellana*	*Roxasellana stellata*	MT527187	15,361	[37]
36			**Cicadulini**	*Cicadula*	*Cicadula* sp.	KX437724	14,929	[30]
37			**Fieberiellini**	*Fieberiella*	*Fieberiella septentrionalis*	MW078430	16,175	[21]
38		**Eurymelinae**	**Macropsini**	*Macropsis*	*Macropsis notata*	NC042723	16,323	[38]
39				*Oncopsis*	*Oncopsis nigrofasciata*	MG813492	15,927	[38]
40		**Coelidiinae**	**Coelidiini**	*Olidiana*	*Olidiana* sp.	KY039119	15,253	DS
41					*Olidiana obliquea*	MN780583	15,312	[39]
42					*Olidiana longisticka*	MN780582	15,993	[39]
43		**Iassinae**	**Batracomorphini**	*Batracomorphus*	*Batracomorphus lateprocessus*	NC045858	15,356	[40]
44			**Krisnini**	*Gessius*	*Gessius rufidorsus*	MN577633	14,634	[40]

*Abbreviation*. *DS* Direct submission, *bp* base pair

### Base composition

The complete mitochondrial genome of three *Abrus* species exhibited heavy AT nucleotide bias, with 76.2%, 76.3%, and 74.7% in *A. daozhenensis, A. yunshanensis,* and *A. expansivus*, respectively. The A+T content of the CR (mean value = 82.4%) was always significantly higher than in other regions, while PCGs showed the lowest A+T content values (mean value = 74.0%) (Table 2). All three species had higher A+T content in rrnL than rrnS, with significantly different mean values (80.8% and 77.9%) (Table 2). All three mitogenomes showed positive AT-skews (0.091 to 0.097) and negative GC-skews (−0.138 to −0.161). Similarly, the control region showed positive AT-skewed (0.011 to 0.041) and negative GC-skewed (−0.103 to −0.119) (Table 2).

**Table 2:** Comparative nucleotide compositions, AT- skews and GC-skews in three *Abrus* species

**Species**	**Whole genome**		**PCGs**		**rrnL**		**rrnS**		**tRNA**		**Control region**	
	**length**	**AT%**	**length**	**AT%**	**length**	**AT%**	**length**	**AT%**	**length**	**AT%**	**length**	**AT%**
***A. daozhenensis***	16391	76.2	10945	74.6	1204	80	745	77.4	1441	76	2035	82.5
***A. yunshanrnsis***	15768	76.3	10945	74.6	1203	80.2	743	78.2	1437	76.2	1947	82.4
***A. expansivus***	15904	74.7	10936	72.9	1204	82.2	756	78.1	1436	75.2	1545	82.2
	**AT-skew**	**GC-skew**	**AT-skew**	**GC-skew**	**AT-skew**	**GC-skew**	**AT-skew**	**GC-skew**	**AT-skew**	**GC-skew**	**AT-skew**	**GC-skew**
***A. daozhenensis***	0.091	-0.144	-0.121	0.008	-0.183	0.2	-0.078	0.168	0.005	0.192	0.011	-0.103
***A. yunshanrnsis***	0.097	-0.161	-0.113	-0.008	-0.177	0.222	-0.097	0.248	0.008	0.202	0.015	-0.119
***A. expansivus***	0.095	-0.138	0.1	-0.151	0.212	-0.269	0.073	-0.187	0.045	0.024	0.041	-0.107

### Gene overlaps, intergenic spacers and non-coding regions

Gene overlaps are present in these three mitogenomes which ranged from 1 bp to 8 bp in length: *A. daozhenensis* (11 gene junctions, 36 bp overlaps), *A. yunshanensis* (12 gene junctions, 36 bp overlaps), and *A. expansivus* (12 gene junctions, 29 bp overlaps). *A. daozhenensis* shares 11 gene overlaps, whereas, *A. yunshanensis* and *A. expansivus*, each with 12 gene overlaps: Ile-Gln (3/3/1 bp), nad2-Trp (8/8/8 bp), Asp-atp8 (7/7/7 bp), atp8-atp6 (-/-/1 bp), Gly-nad3 (2/2/1 bp), Ala-Arg (1/1/1 bp), Arg-Asn (1/1/1 bp), Asn-Ser (4/4/3 bp), Ser-Glu (-/-/1 bp), nad5-His (1/1/- bp), His-nad4 (7/7/4 bp), Pro-nad6 (1/1/1 bp), and cob-Ser (1/1/1 bp) (Supplementary Tables S1–S3).

Intergenic spacers are present in these three mitogenomes which ranged from 1 bp to 39 bp in length: *A. daozhenensis* (7 intergenic spacers), *A. yunshanensis* (8 intergenic spacers), and *A. expansivus* (8 intergenic spacers). The longest intergenic spacers were present between Cys-Tyr with 38, 39, and 18 in *A. daozhenensis*, *A. yunshanensis*, and *A. expansivus*, respectively (Supplementary Tables S1–S3).

The putative control region, or A+T rich region, located between rrnS and trnI, was the most variable region in the whole mitogenome. The full lengths of CR in three *Abrus* mitogenomes were 2,035, 1,947 and 1,545 bp, respectively.

### Transfer RNA and ribosomal RNA genes

For the 22 typical animal tRNA genes in each *Abrus* mitogenome, 14 tRNAs were encoded by the J-strand and the remaining eight were located on the N-strand, ranging from 61 to 76 bp in length. All tRNAs could be folded into the canonical cloverleaf secondary structure except for trnS1 (AGN), which lacks the dihydrouridine (DHU) arm and instead forms a loop in *A. daozhenensis*, *A. yunshanensis* and *A. expansivus* (Supplementary Figs. S1–S3). In addition to the lack of the dihydrouridine (DHU) arm in trnS1 (AGN), *A. expansivus* has a trnG (GGN) that has failed to form a typical clover secondary structure due to the lack of the TѰC arm (Supplementary Fig. S3). Besides the classic A-U and C-G pairs in the secondary structure, there were 35, 36, and 26 G-U base pairings in *A. daozhenensis, A. yunshanensis* and *A. expansivus,* respectively. Some other mismatched base pairs (U-U, A-A, C-U, A-G and A-C) were also found in the acceptor arm and anticodon arm (Supplementary Figs. S1–S3). The large rRNA subunit was located at a conserved position between *trnL1* (CUN) and *trnV*, while the small rRNA subunits was between *trnV* and the control region (Fig. 1A–C). The 16S-rRNA (*rrnL*) gene length are 1,204, 1,203, and 1,204 in *A. daozhenensis*, *A. yunshanensis* and *A. expansivus*, respectively. Whereas 12S-rRNA (*rrnS*) genes with average lengths of 745 bp, 743 bp, and 756 bp, respectively. The mean A+T contents of the two rRNA genes (*rrnL* and *rrnS*) in all three *Abrus* mitogenomes were 80.1% and 77.8%, respectively (Table 2), and *rrn* genes were encoded on the N-strand.

### Protein-coding genes (PCGs), codon usage, and relative synonymous codon usage (RSCU)

The total length of 13 PCG of *A. daozhenensis*, *A. yunshanensis*, and *A. expansivus* are 10,945bp, 10,945bp, and 10,936bp, respectively. Among the 13 protein-coding genes, nine are located on J-strand while the remaining four are on N-strand (Fig. 1A–C). The first codon position had a significantly higher A+T content than the second and third positions (78.1 versus 71.1% and 72.9%) (Supplementary Table 4).

All 13 PCGs started with the standard ATN codons. The starting codons of 13 PCGs are the same in *A. daozhenensis* and *A. yunshanensis*, among which the starting codons of cox1, atp6, cox3, nad4 and cob genes are all ATG, the starting codons of nad2, nad3, nad5, nad4l, and nad6 genes are all ATT, and the starting codons of cox2, atp8, and nad1 genes are all ATA. Except for cox1 and nad6, which started by ATA codon, the remaining starting codons in *A. expansivus* are the same as in *A. daozhenensis* and *A. yunshanensis*. Twelve of the thirteen PCGs in *Abrus* species are terminated with a TAA or TAG codon, except cox2 gene terminates with an incomplete T residue. Except for the cox3 gene that terminated with TAA in *A. daozhenensis* and TAG in *A. yunshanensis*, the stop codons of other genes were the same. Among them, the cox1, atp8, atp6, cox3, nad4, nad4l, nad6, cob, nad1 genes are terminated by standard TAA codon, whereas TAG terminates nad2, nad3, and nad5 genes, and the cox2 gene is terminated with incomplete T as the termination codon. Except for the stop codons of nad3 and nad4l genes in *A. expansivus*, the remaining stop codons are consistent with *A. daozhenensis* and *A. yunshanensis* (Table 3).

**Table 3:** Comparison of length, start and stop codons of 13 protein-coding genes (PCGs) among *Abrus daozhenensis, Abrus yunshanrnsis* and *Abrus expansivus*

**PCGs**	***Abrus daozhenensis***			***Abrus yunshanrnsis***			***Abrus expansivus***		
	**start codon**	**stop codon**	**length (bp)**	**start codon**	**stop codon**	**length (bp)**	**start codon**	**stop codon**	**length (bp)**
nad2	ATT	TAG	975	ATT	TAG	975	ATT	TAG	975
cox1	ATG	TAA	1539	ATG	TAA	1539	ATA	TAA	1539
cox2	ATA	T-	679	ATA	T-	679	ATA	T-	679
atp8	ATA	TAA	153	ATA	TAA	153	ATA	TAA	153
atp6	ATG	TAA	654	ATG	TAA	654	ATG	TAA	654
cox3	ATG	TAA	780	ATG	TAG	780	ATG	TAA	780
nad3	ATT	TAG	354	ATT	TAG	354	ATT	TAA	354
nad5	ATT	TAG	1674	ATT	TAG	1674	ATT	TAG	1674
nad4	ATG	TAA	1308	ATG	TAA	1308	ATG	TAA	1305
nad4l	ATT	TAA	276	ATT	TAA	276	ATT	TAG	276
nad6	ATT	TAA	483	ATT	TAA	483	ATA	TAA	477
cob	ATG	TAA	1137	ATG	TAA	1137	ATG	TAA	1137
nad1	ATA	TAA	933	ATA	TAA	933	ATA	TAA	933

**Fig. 2:**
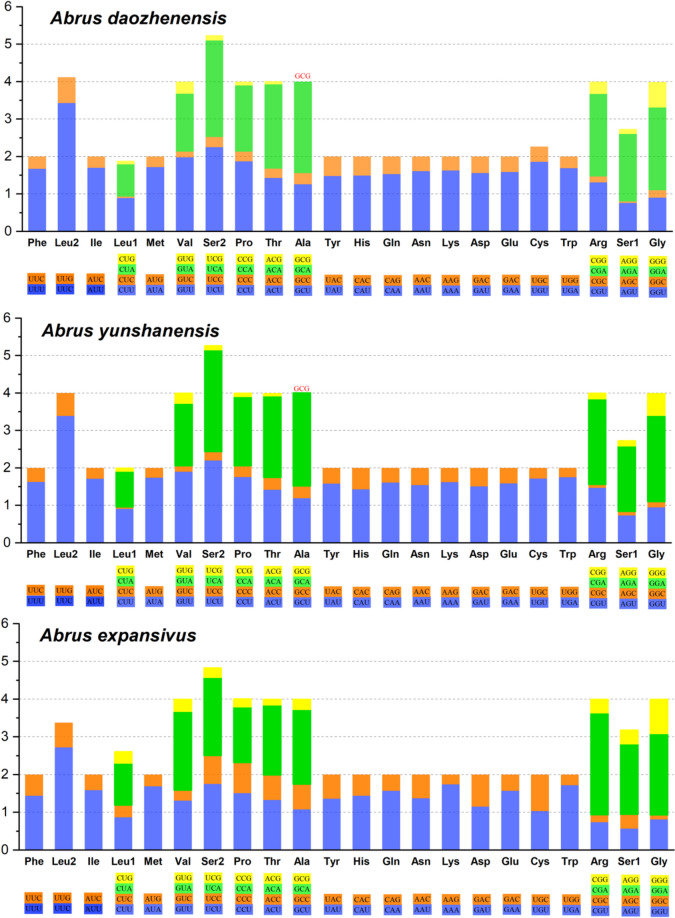
Relative synonymous codon usage (RSCU) of mitochondrial genomes of *Abrus daozhenensis, Abrus yunshanensis* and *Abrus expansivus*. Codons missing in mitogenomes are shown at the top of columns

After excluding the termination codons, the relative synonymous codon usage (RSCU) was calculated and summarized for *A. daozhenensis*, *A. yunshanensis* and *A. expansivus* in Fig. 2. The total numbers of non-stop codons were 3,636, 3,636 and 3,625 in *A. daozhenensis, A. yunshanensis* and *A. expansivus* respectively. The most frequently used amino acids: Isoleucine (Ile) and Methionine (Met), Asparagine (Asn), and Lysine (Lys) were the most frequently used amino acids.

## Phylogenetic relationship

**Fig. 3:**
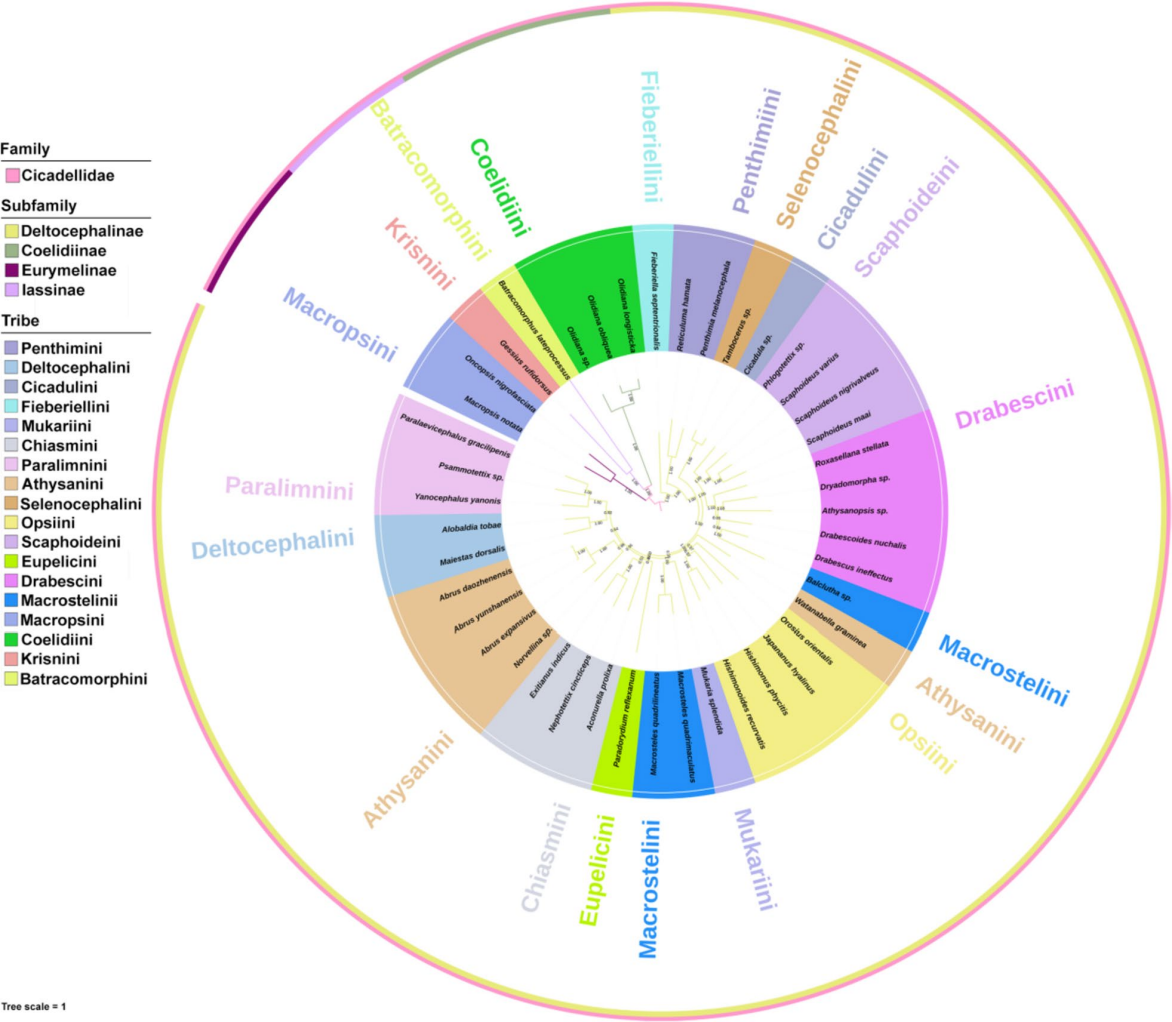
Phylogenetic tree of subfamily Deltocephalinae using the Bayesian inference (BI) analysis method based on the concatenated nucleotide sequences of PCG123 + 2 rRNA + 22 tRNA datasets. Numbers on each node correspond to the posterior probability (PP) values

Previous phylogenetic studies based on morphological and molecular datasets have largely elucidated the relationships among the deltocephaline tribes [14, 18, 19, 26, 29]. The phylogenetic relationships based on 13 PCGs, 2 rRNA genes, and 22 tRNA genes consistently recovered Fieberiellini as sister to the remaining tribes followed Penthimiini and a clad (Selenocephalinithe + Cicadulini), (Scaphoideini + Drabescini + Macrostelini + Drabescini), (Drabescini + Athysanini + Opsiini), (Mukariini + Macrostelini + Eupelicini + Chiasmini), and (Athysanini + Deltocephalini + Paralimnini), in both BI and ML analyses with high support values (Fig. 3, Supplementary Figs. S4-6, S10-12). However, similar combinations of 37 genes with amino acid sequences formed a different clade (Supplementary Figs. S7-9, S13-15). The paraphyly of the following five tribes, Opsiini, Penthimiini, Selenocephalini, Scaphoideini, and Athysanini has not yet been resolved in recent phylogenetic studies based on morphological and molecular datasets. In this study, we recovered the monophyletic Opsiini, Penthimiini, Selenocephalini, Scaphoideini, and Athysanini (except *Watanabella graminea*, previously sequenced species as *Chlorotettix nigromaculatus*, see Zhang & Xing [16] and Yang et al. [24] based on limited available mitogenome sequence data of 37 species. So far, only 14 tribes, 32 genera, and 37 species (including two novel sequences in this study) have been sequenced and analyzed from China and are available on NCBI (https://www.ncbi.nlm.nih.gov/). Phylogenetic trees reconstructed herein based on the BI and ML analyses consistently recovered *Balclutha* sp. (Macrostelini) in tribe Drabescini and *Watanabella graminea* (Athysanini) in Opsiini with high support values (Figs. S4-15).

## Discussion

The goal of this study was to investigate the comparative mitochondrial genome analysis of three *Abrus* species and its phylogenetic position in the tribe Athysanini. The phylogenetic position of Athysanini is still poorly defined, as it includes a large group of polyphyletic genera that have historically been assigned to it mainly because they still exhibit the most typical deltocephaline genitalic and external body characters but lack the distinctive characteristics that other tribes possess. Previous studies based on morphology and molecular datasets consistently recovered a paraphyletic Athysanini [14, 15, 21–23]. Similarly, the paraphyly of the following tribes, Athysanini, Opsiini, Penthimiini, Selenocephalini, and Scaphoideini has not yet been resolved in recent phylogenetic studies [14, 15, 21–23]. In the present study, we recovered the above mentioned tribes as monophylitic, with the exception of Athysanini, in which *Watanabella graminea* was consistently recovered in Opsiini with high strong support values. Further studies must include additional representative species from the above representative tribes to reconstruct the phylogenetic relationships among the tribes in the subfamily Deltocephalinae.

The length of complete mitochondrial genome in insects are remarkable conserved, typically ranging from 14–20 kb in size that encodes 37 genes: 13 PCGs, 2 rRNAs, 22 tRNA, and a non-coding A + T-rich region [3]. With a few exceptions, the length of nearly all known mitochondrial genome sequenced of the subfamily Deltocephalinae ranged from 14-17 kb in size. However, there are few exceptions, in which one or two protein-coding genes are missing (*Dryadomorpha* sp.: KX437736; *Hishimonus phycitis*: KX437727) or with relatively short length of of non-coding A + T-rich region (*Phlogotettix* sp. 2: KX437721; *Cicadulina mbila*: MK251127; *Psammotettix* sp.: KX437742). Herein, we found the consistent genome size in *Abrus* species with previous observations of Deltocephalinae: *A. daozhenensis* (GenBank: MZ274046), *A. yunshanensis* (GenBank: MZ274047), and *A. expansivus* (GenBank: MK033020) are 16,391 bp, 15,768 bp, and 15,904 bp in size, respectively (Table 1). The variation in mitogenome size among the different deltocephaline species is mainly due to the variable number of repeats in the control region. The mitogenome sequences of *Abrus* species were highly conserved in gene content, gene size, gene order, base composition, codon usage of PCGs and tRNA secondary structures.

Comparative studies of leafhopper mitochondrial genomes suggest that genome rearrangements are very rare, and was proposed as a genus-level trait [32]. All *Abrus* species from this study, and the other deltocephaline species sequenced so far are arranged in the putative ancestral insect gene arrangement, tRNA cluster trnW-trnC-trnY. So far, only three leafhopper species, *Macrosteles quadrilineatus* [31] and *Macrosteles quadrimaculatus* [32] shares the same tRNA rearrangement, that tRNA cluster trnW-trnC-trnY is rearranged to trnC-trnW-trnY. Species *Japananus hyalinus* are rearranged tRNA cluster trnY-trnW-trnC [29]. Complete mitogenome sequences for additional Deltocephaline specimens are needed to facilitate broader comparison and to identify features of the potential novel rearrangements in the gene order of the insects. The mitogenome of leafhoppers is a typical conserved circular, about 14.5–17 kb in length which shows the conserved gene structures, it contains double-stranded DNA molecule, 37 typical mitochondrial genes, and does not have introns. Although conservative, the novel rearrangements of the mitogenomes allowed us to identify with enough variation for population genetic or phylogeographic studies.

## Conclusions

The present phylogenetic analyses based on 12 phylogenetic trees based on the various combination of concatenated datasets of 13 PCGs, 2 rRNA genes, and 22 tRNA genes along with different partition datasets and models strongly supported for paraphyletic Athysanini in both BI and ML methods using the six concatenated datasets: amino acid sequences and nucleotides from different combinations of protein-coding genes (PCGs), ribosomal RNA (rRNAs), and transfer RNA (tRNAs). In all analyses, Fieberiellini is consistently recovered as sister to the remaining tribes, however, the internal tribal relationships by using the PCGs and amino acid sequences formed a different clade. The unresolve paraphyly of the following five tribes, Opsiini, Penthimiini, Selenocephalini, Scaphoideini, and Athysanini have recovered the monophyletic, except Athysanini with *Watanabella graminea*, previously sequenced species as *Chlorotettix nigromaculatus*, consistently recovered in Opsiini with high support values.

In this study, we newly sequenced and analyzed the complete mitochondrial genomes of two *Abrus* species and compared them with the published mitogenome of *A. expansivus* Xing & Li, 2014. Comparative mitogenome analyses showed that the gene content, gene order, gene size, nucleotide base composition, codon usage of PCGs, and tRNA secondary structures are highly conserved in *Abrus* species. The complete mitochondrial genome in *Abrus* species is around 10,936-10,945 bp. The full lengths of CR in *Abrus* species range from 2,035bp in *A. daozhenensis* to 15,45bp in *A. expansivus*. All PCGs in *Abrus* species were initiated with ATN codons, however, 12 of the 13 PCGs are terminated with a TAA or TAG codon, except cox2 gene terminates with an incomplete T residue. All 22 tRNA genes had typical cloverleaf secondary structures, except for *trnS1* (AGN), which lacks the dihydrouridine arm, and distinctively *trnG* in the mitogenome of *A. expansivus* lacks the TψC arm. At present, *Abrus* belongs to the tribe Athysanini based on both morphological and molecular datasets, which is strongly supported in present phylogenetic analyses in both BI and ML methods using the six concatenated datasets: amino acid sequences and nucleotides from different combinations of protein-coding genes (PCGs), ribosomal RNA (rRNAs), and transfer RNA (tRNAs).

## Materials and methods

### Taxon sampling

The adult specimens of *Abrus daozhenensis* and *A. yunshanensis* were collected with a hand-net from the bamboo plant at the Kuankuoshui National Nature Reserve, Guiyang, Guizhou, China, from June to August 2020. The permission was taken from Kuankuoshui National Nature Reserve to collect these leaf hopper species. The field-collected specimens were initially preserved in 100% ethanol which was later identified based on morphological characters following Chen et al. [11] and Xing & Li [13] and stored at − 20 °C until DNA extraction. Vouchers were deposited at the Institute of Entomology, Guizhou University, Guiyang, China.

### DNA extraction and sequencing

The entire body of *A. daozhenensis* and *A. yunshanensis* were sent to Guangzhou Ruike Gene Technology Co. (China) for genomic DNA extraction and sequencing. Total genomic DNA was extracted from the thoracic muscle tissues and legs of each individual using the DNeasy© Tissue Kit (Qiagen) according to the manufacturer's protocol. For Illumina sequencing, genomic DNA was isolated using TIANamp Genomic DNA Kit (Tiangen, China). The Illumina sequencing library was generated using Truseq Nano DNA HT Sample Prep Kit (Illumina, USA). The complete mitogenome was sequenced using high-throughput sequencing on the Illumina Novaseq 6000 platform with an average insert size of 350 bp and a paired-end 150 bp (PE 150) sequencing strategy to generate sequencing data not less than 2 GB. Raw reads were trimmed of adapters using Trimmomatic [41].

### Sequence assembly, annotation and analyses

In order to investigate the phylogenetic relationships among the deltocephaline tribes, we retrieved the GenBank file of almost all available mitogenomic data of deltocephaline tribes (14 out of 39 currently recognized tribes), including 37 species in 32 genera as ingroup. Outgroup sampling comprised eight species in three subfamilies: Eurymelinae, Coelidiinae, and Iassinae (Table 1). Consensus sequences of *A. daozhenensis* and *A. yunshanensis* were assembled with the complete mitochondrial genome of *A. expansivus* (NC_045238) as a reference in GENEIOUS v. 10.2.3 (https://www.geneious.com/) [42]. Both the assembled sequences were aligned and compared with the published sequence of Deltocephalinae to extract the 13 PCG and 2 rRNA genes by using MUSCLE [43] in MEGA 7.0 [44]. The 22 transfer RNA (tRNA) genes were annotated using the MITOS web server (http://mitos.bioinf.unileipzig.de/index.py) [45]. The secondary structure of tRNA was obtained from the MITOS web server and manually edited in Adobe Photoshop CS 6.0 (Figs. S1–S3). The graphical map of the circular genome and annotation was made using the CGView Server (http://stothard.afns.ualberta.ca/cgview_server/) [46]. MEGA Version 7.0 was used to analyze the base composition of the complete mitogenome. The strand asymmetry was calculated by using the formulas: GC-skew = [(G – C)/(G + C)] and AT-skew = [(A – T)/(A + T)] [47].

### Phylogenetic analysis

For phylogenetic analyses, the concatenated nucleotide and amino acid sequences of all 13 PCGs, 22 tRNA genes, and 2 rRNA genes were extracted using PhyloSuite (v1.2.3) [48] and aligned with the codon and Normal mode using the invertebrate mitochondrial code and automatic strategy in MAFFT [49]. The aligned PCGs were then refined using MACSE and trimmed by GBlock. The aligned RNA genes and amino acid sequences were trimmed by trimAl. Aligned genes were concatenated to generate six datasets using PhyloSuite: (1) PCG123 (nucleotide data: 1,067bp); (2) PCG123 + 2 rRNA (nucleotide data: 12,521bp); (3) PCG123 + 22 tRNA + 2 rRNA (nucleotide data: 13,904bp); (4) PCG_AA (amino acid sequences: 3,572bp); (5) PCG_AA + 2 rRNA (amino acid sequences: 5,422bp); and PCG_AA + 2 rRNA + 22 tRNA (amino acid sequences: 6,805bp). We used PartitionFinder v.2.1.1 [50] to determine the best partitioning schemes for the datasets under the Bayesian Information Criterion (BIC). Detailed information on the best partitioning schemes and substitution model selection for ML and BI analyses using PartionFinder2 and ModelFinder are summarized in Supplementary Tables S5-S16. The phylogenetic trees were reconstructed using two methods: Bayesian inference (BI) and Maximum Likelihood (ML) based on various combinations of nucleotide and amino acid sequences. BI analyses were conducted using MrBayes v.3.2.7a [51] implemented in PhyloSuite software with various data partition schemes and best-fitting models determined by PartitionFinder, and Maximum Likelihood (ML) was performed on the IQ-tree using ultrafast bootstrap with 5000 replicates as implemented on the website server (http://iqtree.cibiv.univie.ac.at). The BI analyses contains four simultaneous Markov chain Monte Carlo (MCMC) runs of 2 million generations, and sampled every 1000 generations. The initial 25% of the sampled data were discarded as burn-in. Other parameters were kept at default settings. The finalized trees were visualized and edited with FIGTREE v1.3.1 [52] and the Interactive Tree of Life (iTOL: https://itol.embl.de) version 5 [53].

### Supplementary Information

**Additional file 1:** **Supplementary Figure S1.** Inferred secondary structures of 22 tRNA genes in the mitochondrial genome of *Abrus daozhenensis*. Watson-Crick base pairings are illustrated by lines (-), whereas GU base pairings are illustrated by dots (·).**Supplementary Figure S2.** Inferred secondary structures of 22 tRNA genes in the mitochondrial genome of *Abrus yunshanensis*. Watson-Crick base pairings are illustrated by lines (-), whereas GU base pairings are illustrated by dots (·).**Supplementary Figure S3.** Inferred secondary structures of 22 tRNA genes in the mitochondrial genome of *Abrus expansivus*. Watson-Crick base pairings are illustrated by lines (-), whereas GU base pairings are illustrated by dots (·).**Supplementary Figure S4.** Phylogenetic tree produced by Bayesian inference analysis of the PCG123 datasets. Numbers at nodes are Bayesian posterior probability (BPP) support values. **Supplementary Figure S5.** Phylogenetic tree produced by Bayesian inference analysis of the PCG123 + 2 rRNA datasets. Numbers at nodes are Bayesian posterior probability (BPP) support values. **Supplementary Figure S6.** Phylogenetic tree produced by Bayesian inference analysis of the PCG123 + 2 rRNA + 22 tRNA datasets. Numbers at nodes are Bayesian posterior probability (BPP) support values. **Supplementary Figure S7.** Phylogenetic tree produced by Bayesian inference analysis of the PCG123_AA dataset. Numbers at nodes are Bayesian posterior probability (BPP) support values. **Supplementary Figure S8.** Phylogenetic tree produced by Bayesian inference analysis of the PCG123_AA + 2 rRNA datasets. Numbers at nodes are Bayesian posterior probability (BPP) support values. **Supplementary Figure S9.** Phylogenetic tree produced by Bayesian inference analysis of the PCG123_AA + 2 rRNA + 22 tRNA datasets. Numbers at nodes are Bayesian posterior probability (BPP) support values. **Supplementary Figure S10.** Phylogenetic tree produced by maximum likelihood analyses based on PCG123 datasets. Numbers at nodes are bootstrap support values (BS). **Supplementary Figure S11.** Phylogenetic tree produced by maximum likelihood analyses based on PCG123+ 2 rRNA datasets. Numbers at nodes are bootstrap support values (BS). **Supplementary Figure S12.** Phylogenetic tree produced by maximum likelihood analyses based on PCG123+ 2 rRNA + 22 tRNA datasets. Numbers at nodes are bootstrap support values (BS). **Supplementary Figure S13.** The phylogenetic tree produced by maximum likelihood analyses based on PCG123_AA datasets. Numbers at nodes are bootstrap support values (BS). **Supplementary Figure S14.** The phylogenetic tree produced by maximum likelihood analyses based on PCG123_AA+ 2 rRNA datasets. Numbers at nodes are bootstrap support values (BS). **Supplementary Figure S15.** The phylogenetic tree produced by maximum likelihood analyses based on PCG123_AA+ 2 rRNA + 22 tRNA datasets. Numbers at nodes are bootstrap support values (BS).

**Additional file 2:** **Supplementary Table S1.** Organization of the *Abrus daozhenensis* mitochondrial genome. **Supplementary Table S2.** Organization of the *Abrus yunshanensis* mitochondrial genome. **Supplementary Table S3.** Organization of the *Abrus expansivus* mitochondrial genome. **Supplementary Table S4.** Overall A+T content in the first (P1), second (P2) and third (P3) codon positions. **Supplementary Table S5.** The best partitioning schemes and models for the Bayesian inference (BI) method based on 123PCG dataset selected by PartitionFinder. **Supplementary Table S6.** The best partitioning schemes and models for the Bayesian inference (BI) method based on 123PCG + 2 rRNA dataset selected by PartitionFinder. **Supplementary Table S7.** The best partitioning schemes and models for the Bayesian inference (BI) method based on 123PCG + 2 rRNA + 22 tRNA dataset selected by PartitionFinder. **Supplementary Table S8.** The best partitioning schemes and models for the Bayesian inference (BI) method based on 123PCG_AA dataset selected by PartitionFinder. **Supplementary Table S9. **The best partitioning schemes and models for the Bayesian inference (BI) method based on 123PCG_AA + 2 rRNA dataset selected by PartitionFinder. **Supplementary Table S10. **The best partitioning schemes and models for the Bayesian inference (BI) method based on 123PCG_AA + 2 rRNA + 22 tRNA dataset selected by PartitionFinder. **Supplementary Table S11. **The best partitioning schemes and models for maximum-likelihood (ML) analyses on 123PCG dataset selected by PartitionFinder. **Supplementary Table S12. **The best partitioning schemes and models for maximum-likelihood (ML) analyses on 123PCG + 2rRNA dataset selected by PartitionFinder. **Supplementary Table S13. **The best partitioning schemes and models for maximum-likelihood (ML) analyses on 123PCG + 2rRNA + 22tRNA dataset selected by PartitionFinder. **Supplementary Table S14.** The best partitioning schemes and models for maximum-likelihood (ML) analyses on 123PCG_AA dataset selected by PartitionFinder. **Supplementary Table S15. **The best partitioning schemes and models for maximum-likelihood (ML) analyses on 123PCG_AA + 2rRNA dataset selected by PartitionFinder. **Supplementary Table S16. **The best partitioning schemes and models for maximum-likelihood (ML) analyses on 123PCG_AA + 2rRNA + 22tRNA dataset selected by PartitionFinder.

## Data Availability

The mitogenome sequences of *Abrus daozhenensis* Chen, Yang & Li, 2012 (GenBank accession no: MZ274046), and *Abrus expansivus* Xing & Li, 2014 (GenBank: MK033020) and *A. yunshanensis* Chen, Yang & Li, 2012 (GenBank accession no: MZ274047) have been deposited on GenBank.
