# Supplementary material for: Comparative mitochondrial genome analysis of three leafhopper species of the genus Abrus Dai & Zhang (Hemiptera: Cicadellidae: Deltocephalinae) from China with phylogenetic implication
Source: BMC Genomics. 2023 Nov 27;24:714. doi: 10.1186/s12864-023-09809-0 (PMC10680345; doi:10.1186/s12864-023-09809-0)
Supplement: Supplementary file 1 — Additional file 1: Supplementary Figure S1. Inferred secondary structures of 22 tRNA genes in the mitochondrial genome of Abrus daozhenensis. Watson-Crick base pairings are illustrated by lines (-), whereas GU base pairings are illustrated by dots (·).Supplementary Figure S2. Inferred secondary structures of 22 tRNA genes in the mitochondrial genome of Abrus yunshanensis. Watson-Crick base pairings are illustrated by lines (-), whereas GU base pairings are illustrated by dots (·).Supplementary Figure S3. Inferred secondary structures of 22 tRNA genes in the mitochondrial genome of Abrus expansivus. Watson-Crick base pairings are illustrated by lines (-), whereas GU base pairings are illustrated by dots (·).Supplementary Figure S4. Phylogenetic tree produced by Bayesian inference analysis of the PCG123 datasets. Numbers at nodes are Bayesian posterior probability (BPP) support values. Supplementary Figure S5. Phylogenetic tree produced by Bayesian inference analysis of the PCG123 + 2 rRNA datasets. Numbers at nodes are Bayesian posterior probability (BPP) support values. Supplementary Figure S6. Phylogenetic tree produced by Bayesian inference analysis of the PCG123 + 2 rRNA + 22 tRNA datasets. Numbers at nodes are Bayesian posterior probability (BPP) support values. Supplementary Figure S7. Phylogenetic tree produced by Bayesian inference analysis of the PCG123_AA dataset. Numbers at nodes are Bayesian posterior probability (BPP) support values. Supplementary Figure S8. Phylogenetic tree produced by Bayesian inference analysis of the PCG123_AA + 2 rRNA datasets. Numbers at nodes are Bayesian posterior probability (BPP) support values. Supplementary Figure S9. Phylogenetic tree produced by Bayesian inference analysis of the PCG123_AA + 2 rRNA + 22 tRNA datasets. Numbers at nodes are Bayesian posterior probability (BPP) support values. Supplementary Figure S10. Phylogenetic tree produced by maximum likelihood analyses based on PCG123 datasets. Numbers at nodes are boo [file 12864_2023_9809_MOESM1_ESM.docx]

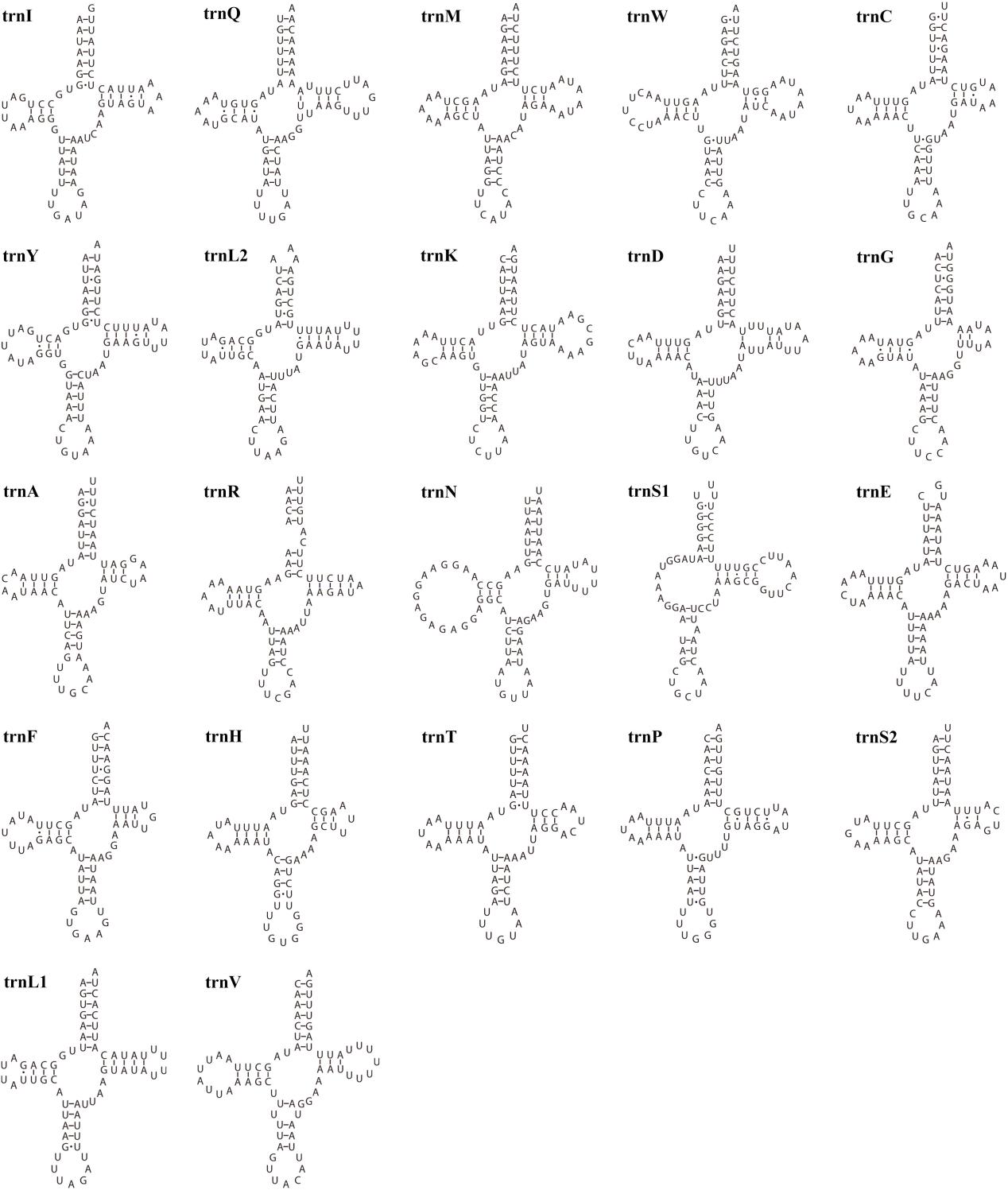


**Supplementary Figure S1.** Inferred secondary structures of 22 tRNA genes in the mitochondrial genome of *Abrus daozhenensis*. Watson-Crick base pairings are illustrated by lines (-), whereas GU base pairings are illustrated by dots (·).


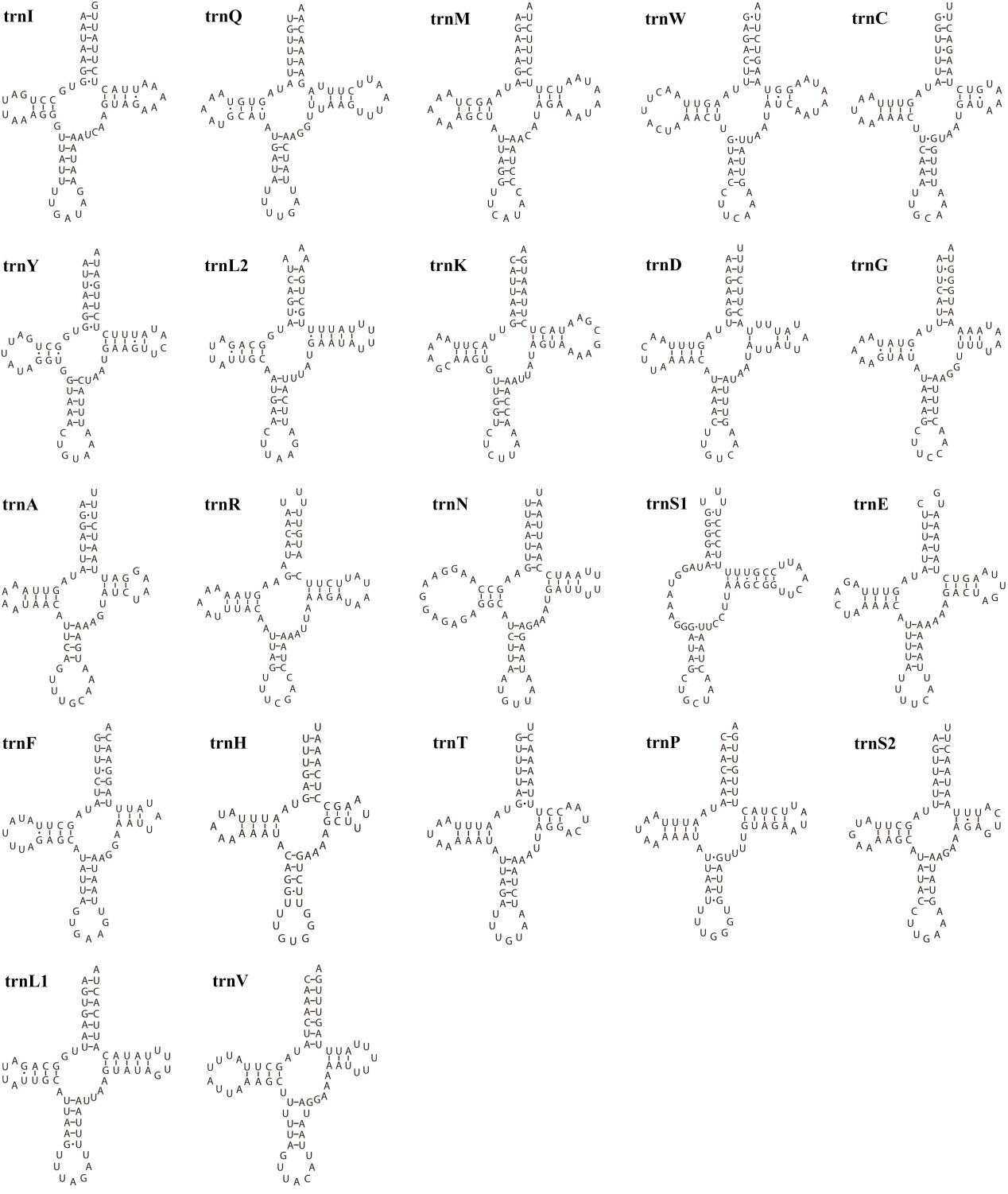


**Supplementary Figure S2.** Inferred secondary structures of 22 tRNA genes in the mitochondrial genome of *Abrus yunshanensis*. Watson-Crick base pairings are illustrated by lines (-), whereas GU base pairings are illustrated by dots (·).


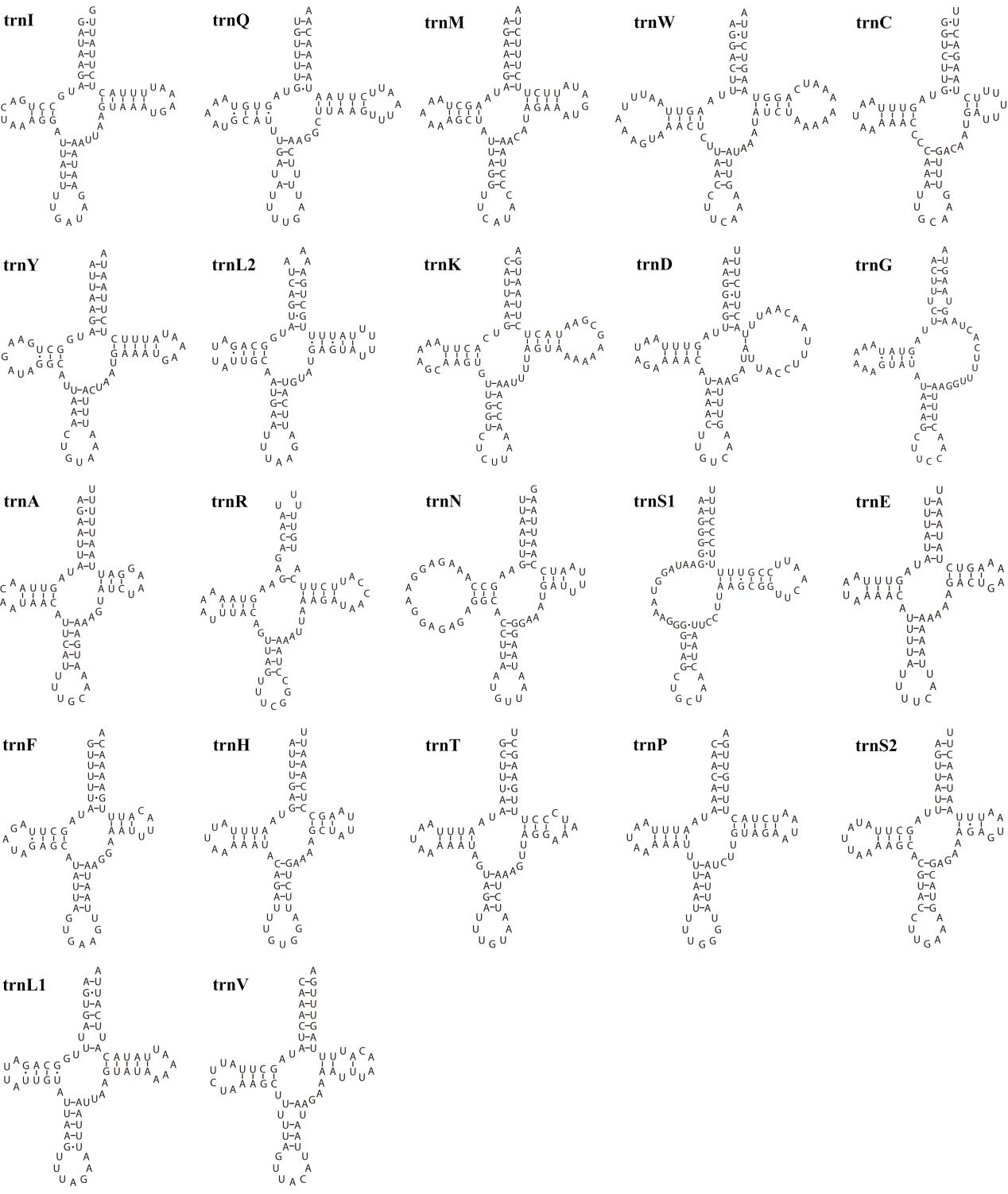


**Supplementary Figure S3.** Inferred secondary structures of 22 tRNA genes in the mitochondrial genome of *Abrus expansivus*. Watson-Crick base pairings are illustrated by lines (-), whereas GU base pairings are illustrated by dots (·).


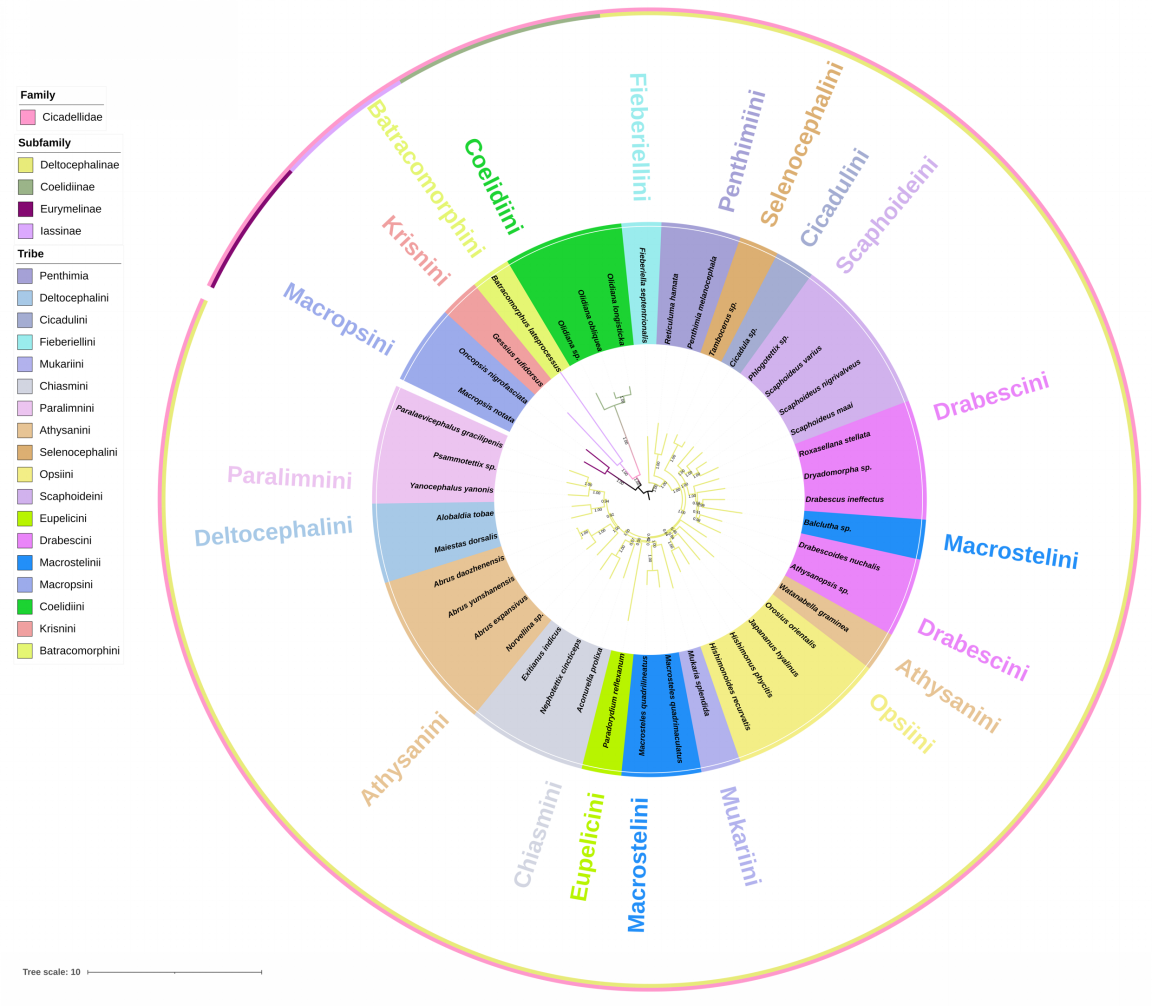
**Supplementary Figure S4.** Phylogenetic tree produced by Bayesian inference analysis of the PCG123 datasets. Numbers at nodes are Bayesian posterior probability (BPP) support values.


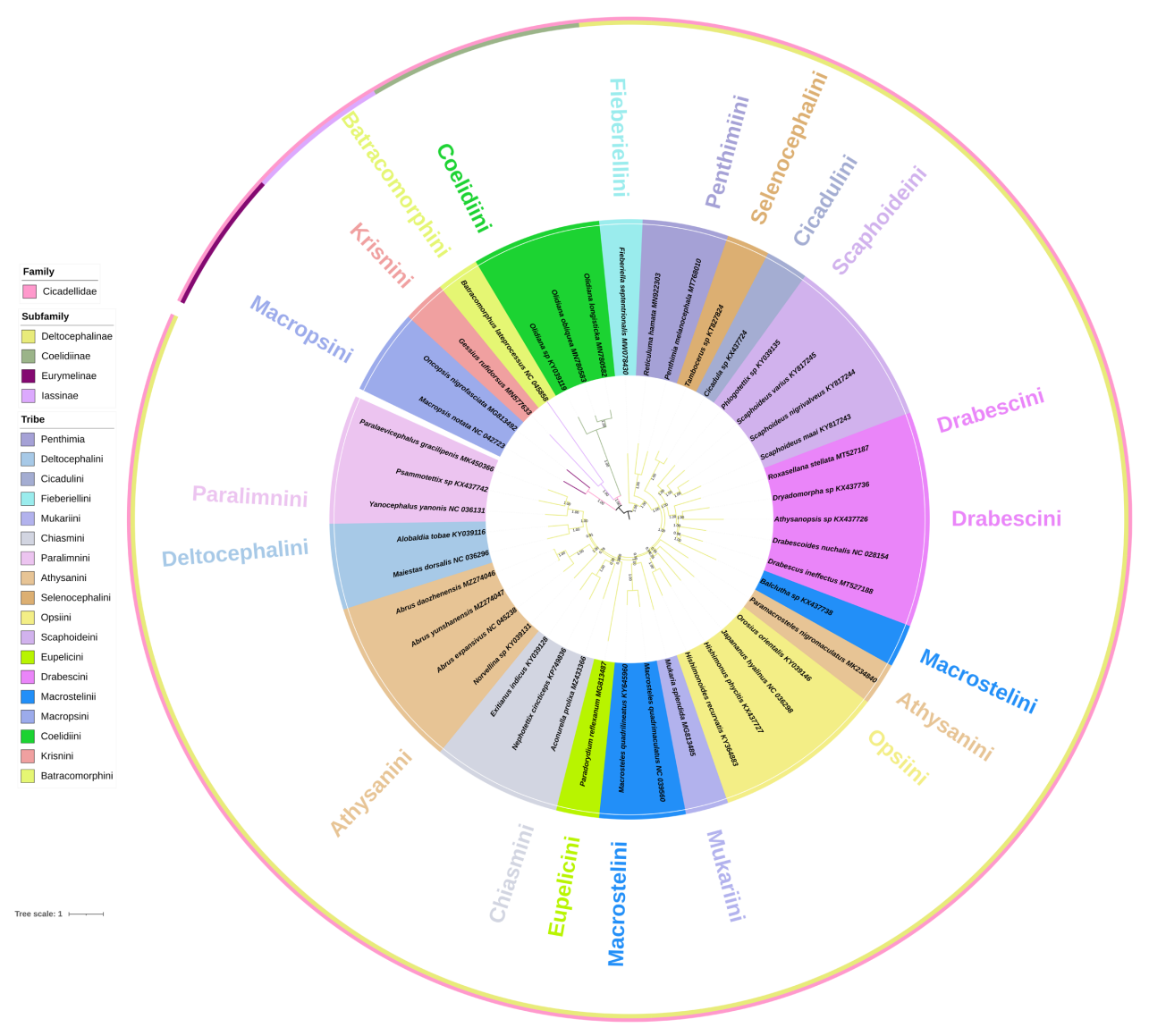
**Supplementary Figure S5.** Phylogenetic tree produced by Bayesian inference analysis of the PCG123 + 2 rRNA datasets. Numbers at nodes are Bayesian posterior probability (BPP) support values.


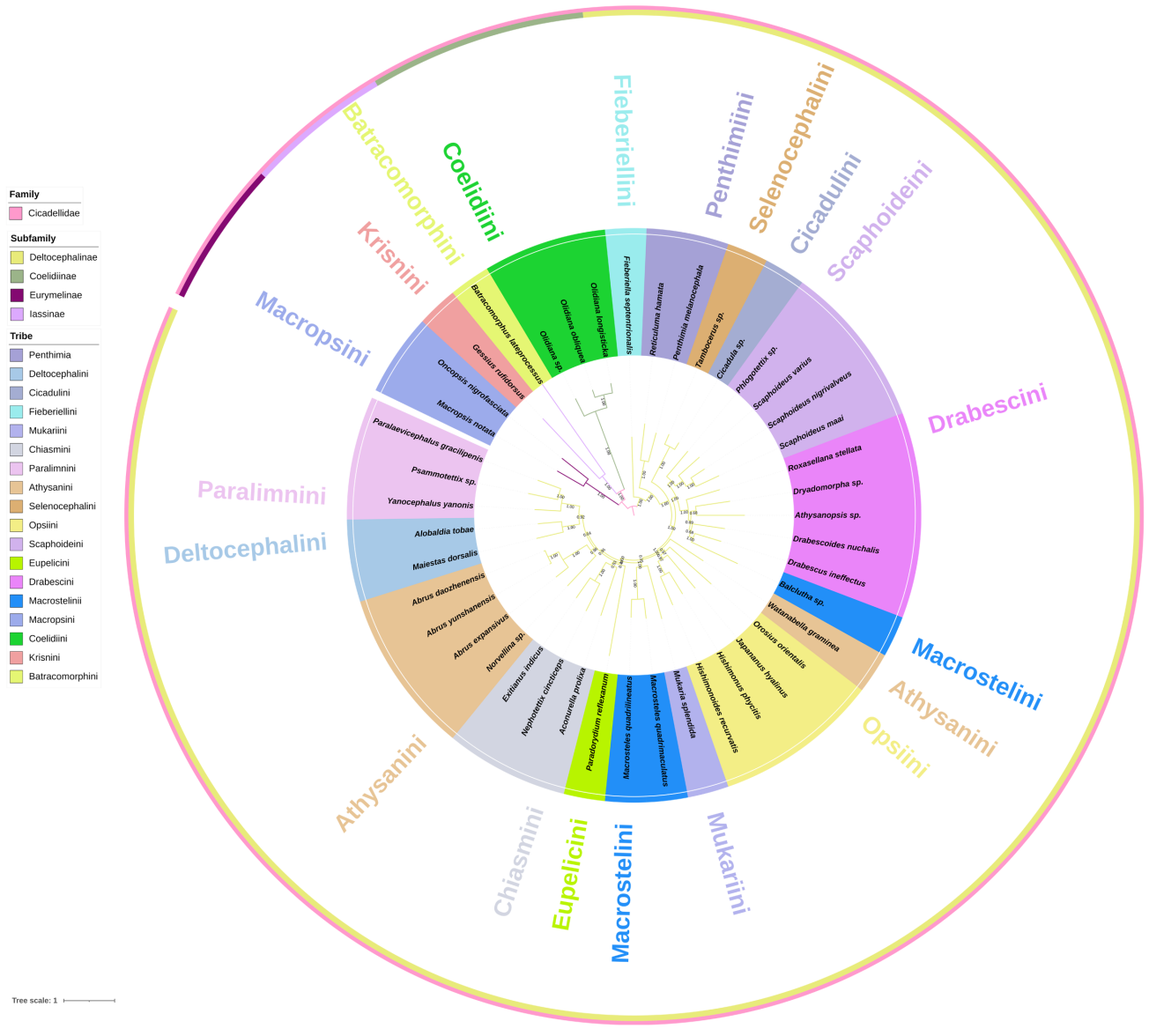
**Supplementary Figure S6.** Phylogenetic tree produced by Bayesian inference analysis of the PCG123 + 2 rRNA + 22 tRNA datasets. Numbers at nodes are Bayesian posterior probability (BPP) support values.


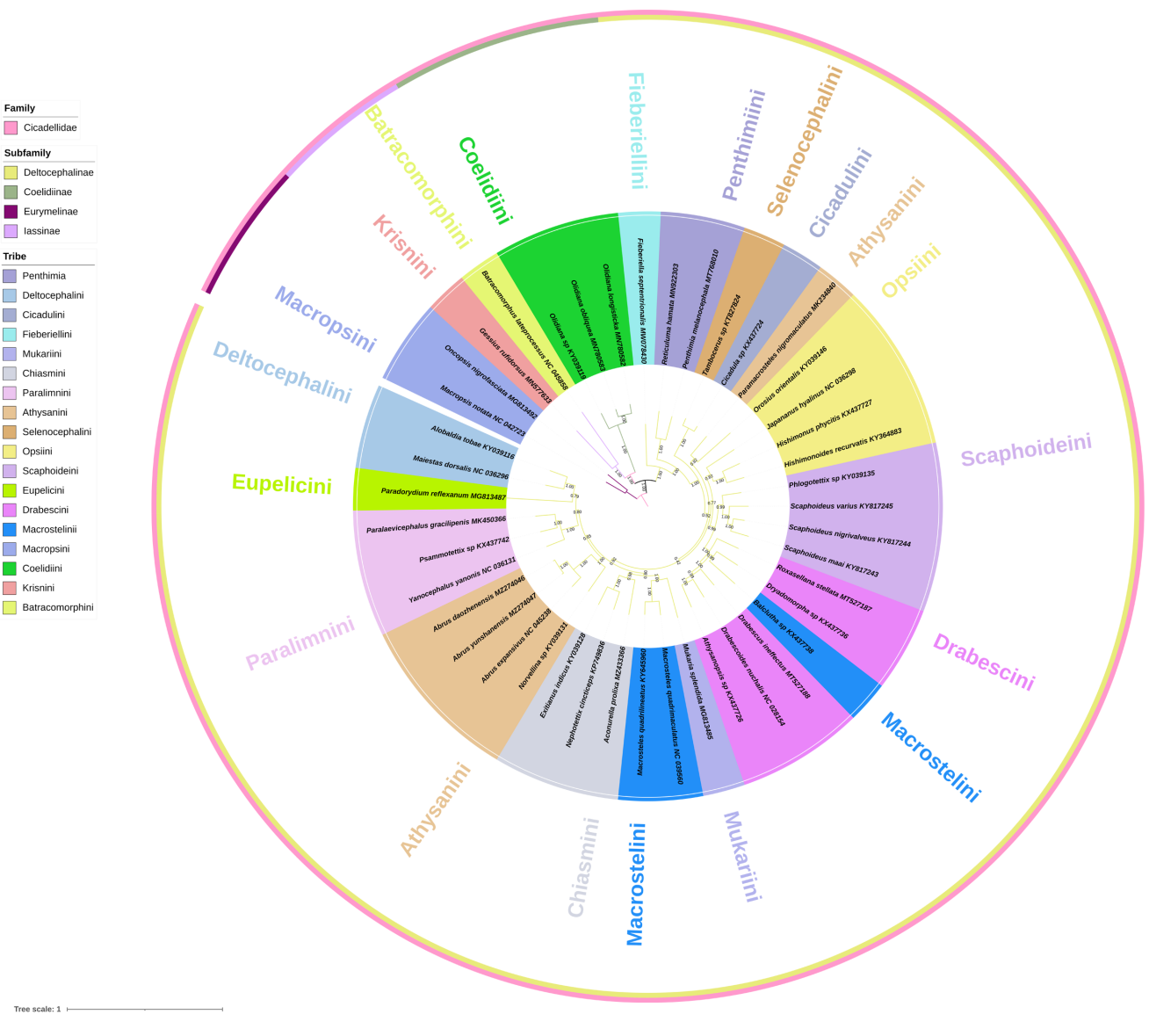
**Supplementary Figure S7.** Phylogenetic tree produced by Bayesian inference analysis of the PCG123_AA dataset. Numbers at nodes are Bayesian posterior probability (BPP) support values.


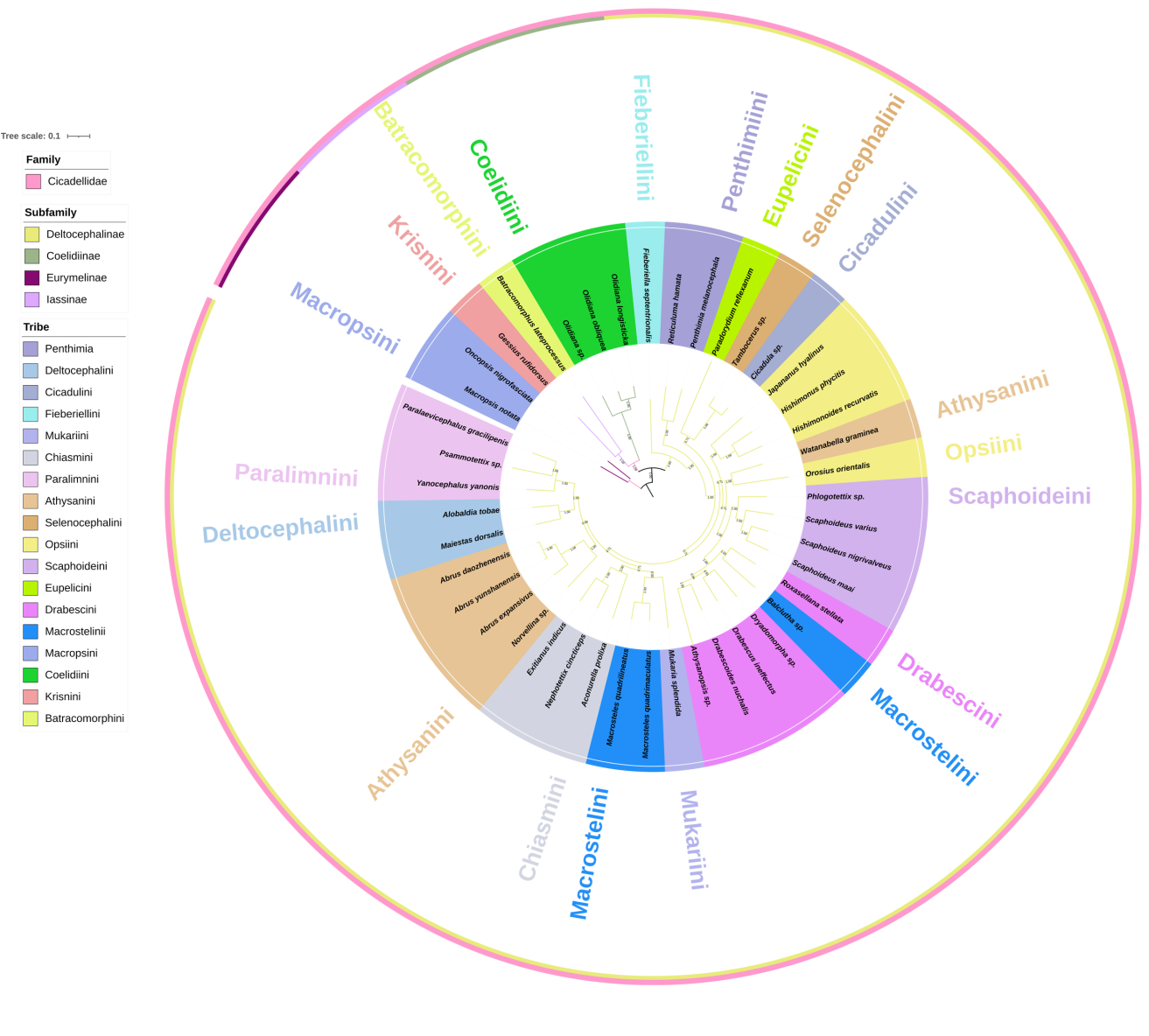


**Supplementary Figure S8.** Phylogenetic tree produced by Bayesian inference analysis of the PCG123_AA + 2 rRNA datasets. Numbers at nodes are Bayesian posterior probability (BPP) support values.


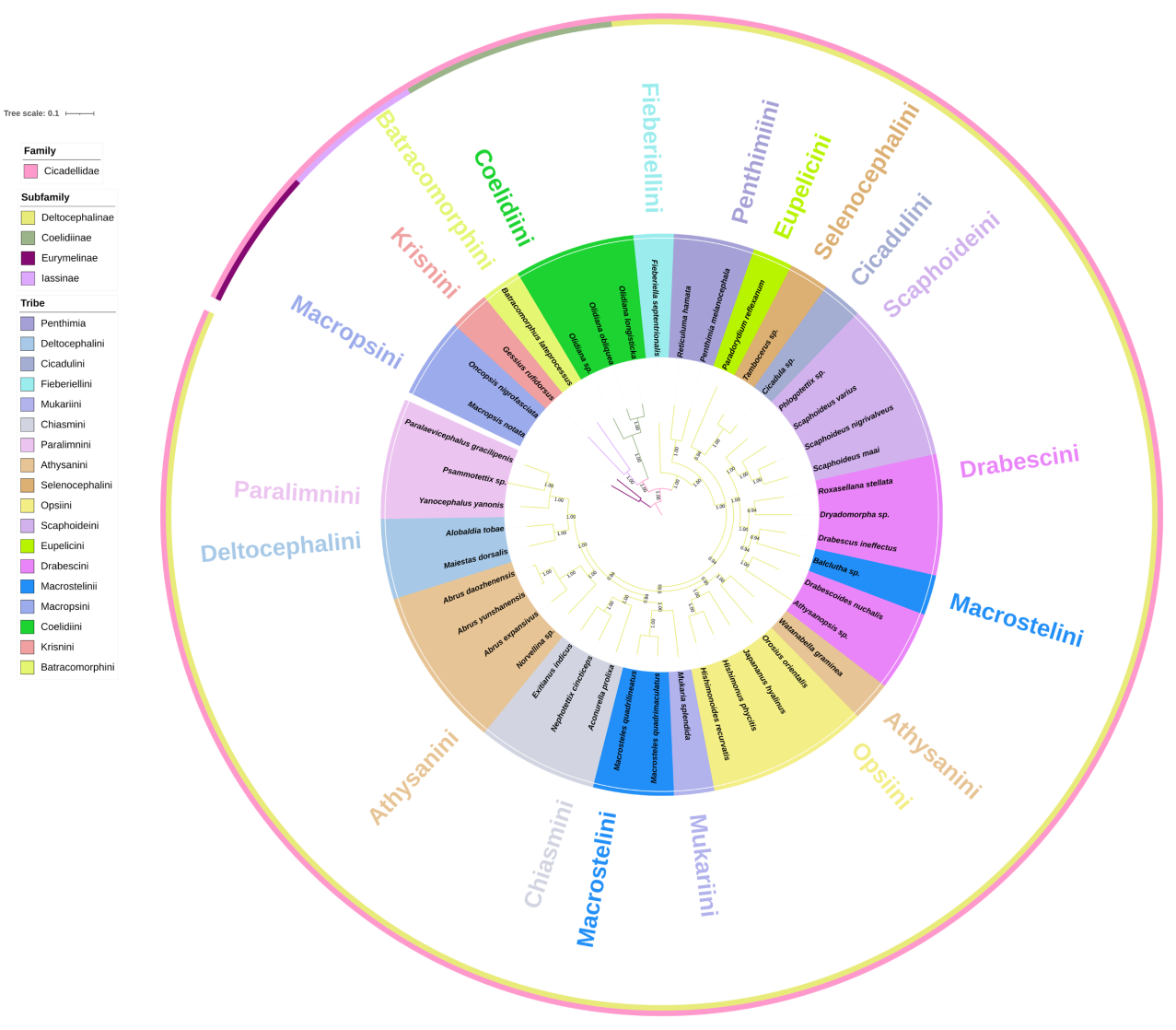
**Supplementary Figure S9.** Phylogenetic tree produced by Bayesian inference analysis of the PCG123_AA + 2 rRNA + 22 tRNA datasets. Numbers at nodes are Bayesian posterior probability (BPP) support values.


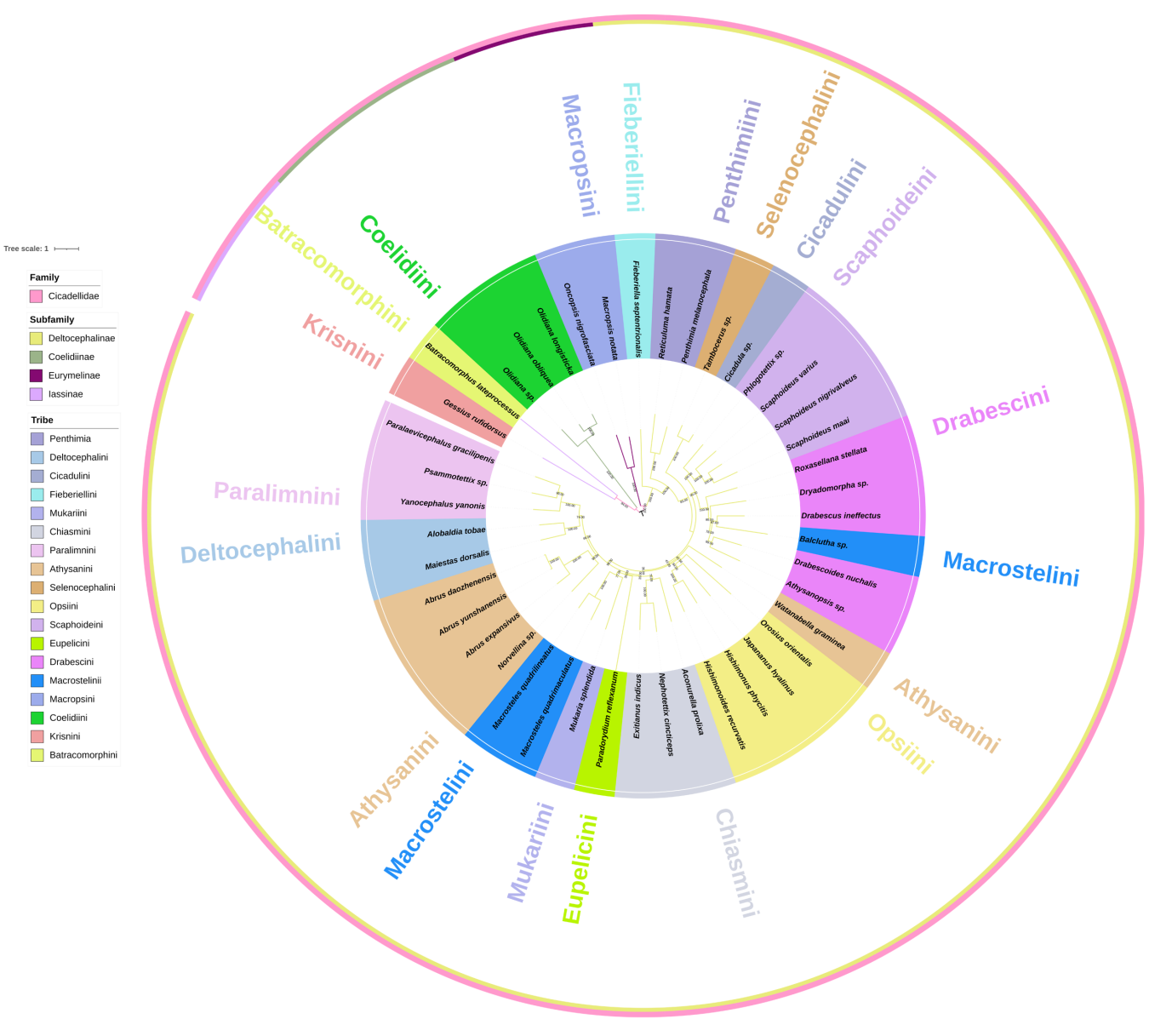
**Supplementary Figure S10.** Phylogenetic tree produced by maximum likelihood analyses based on PCG123 datasets. Numbers at nodes are bootstrap support values (BS).


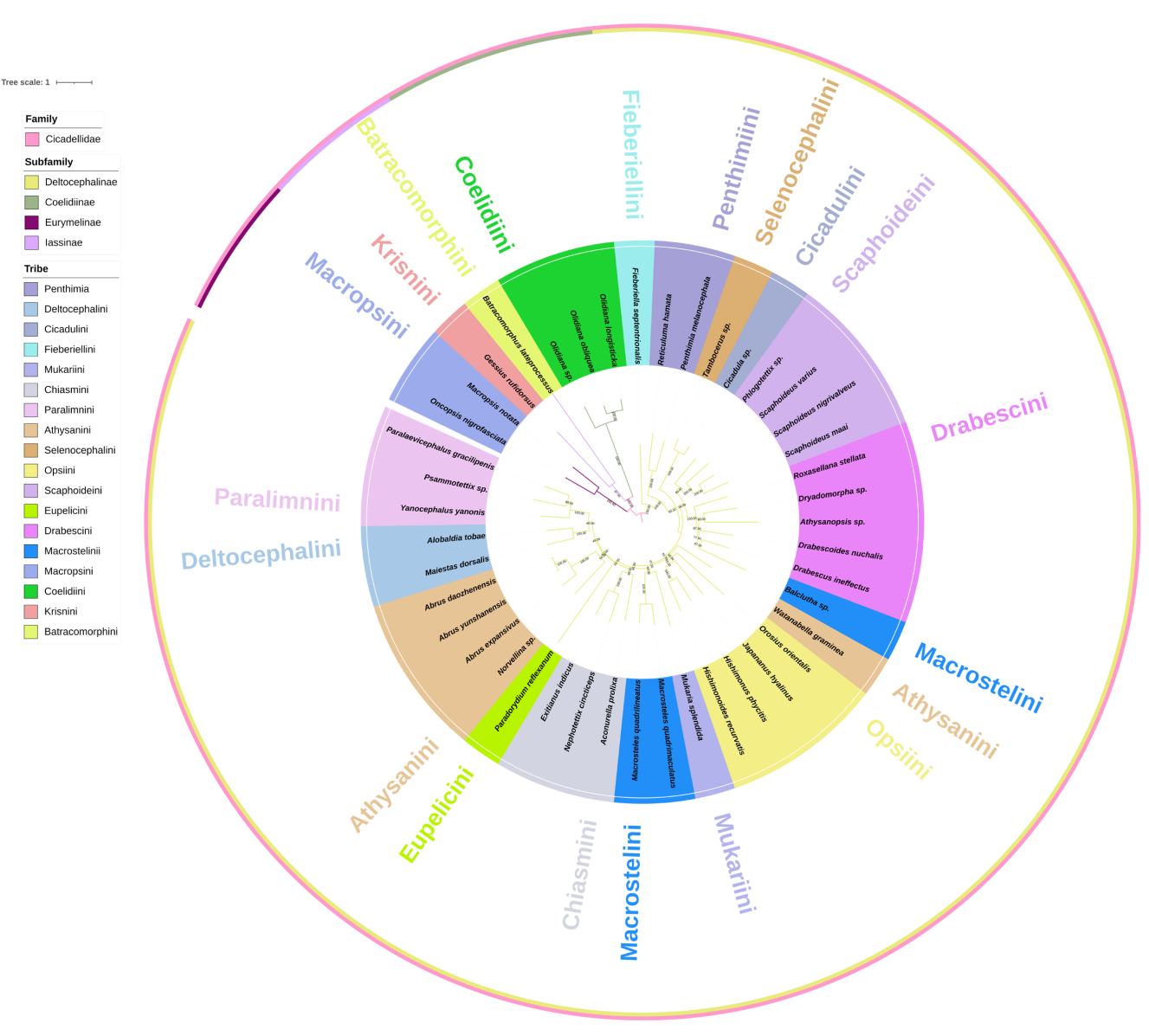


**Supplementary Figure S11.** Phylogenetic tree produced by maximum likelihood analyses based on PCG123+ 2 rRNA datasets. Numbers at nodes are bootstrap support values (BS).


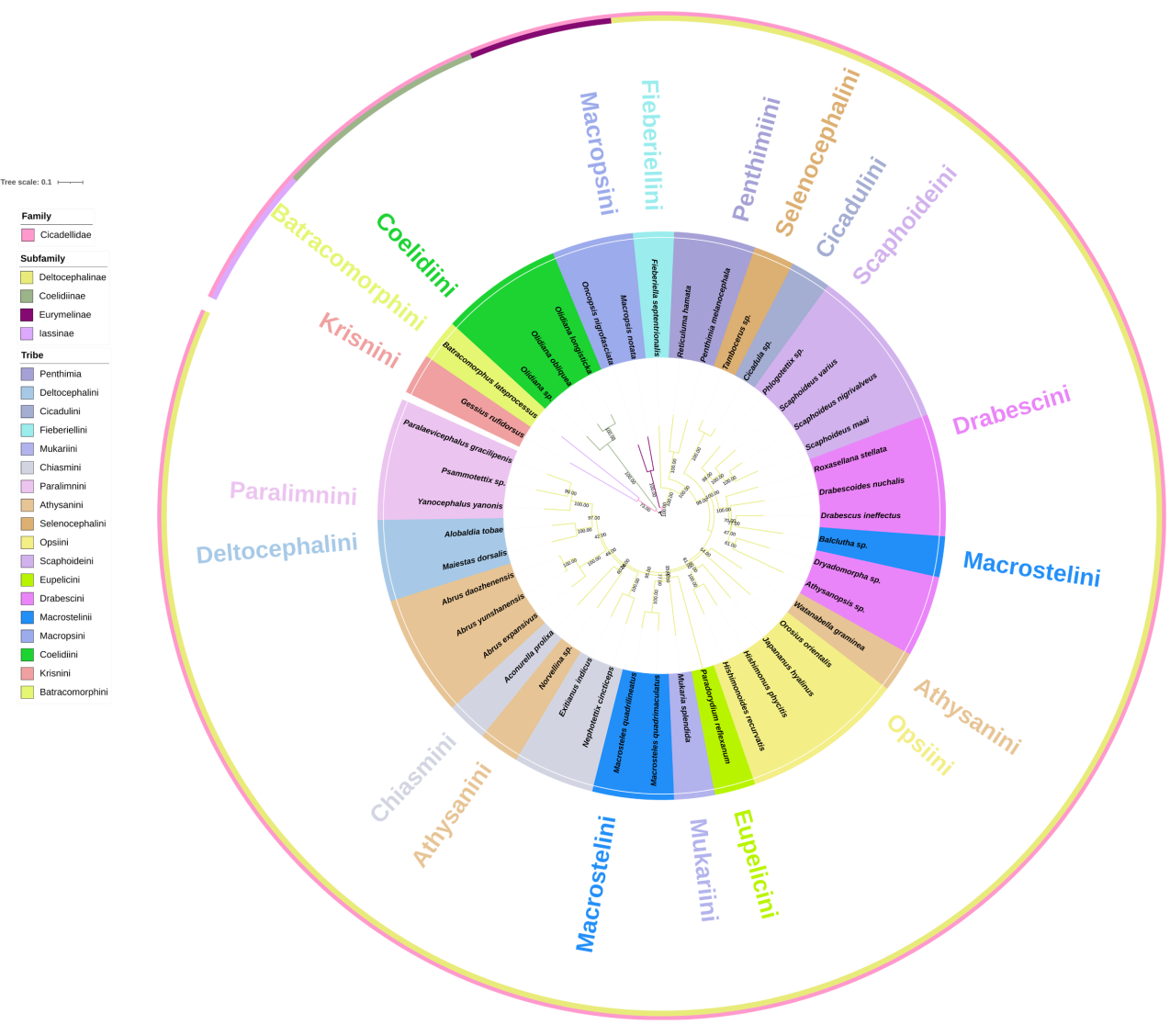
**Supplementary Figure S12.** Phylogenetic tree produced by maximum likelihood analyses based on PCG123+ 2 rRNA + 22 tRNA datasets. Numbers at nodes are bootstrap support values (BS).


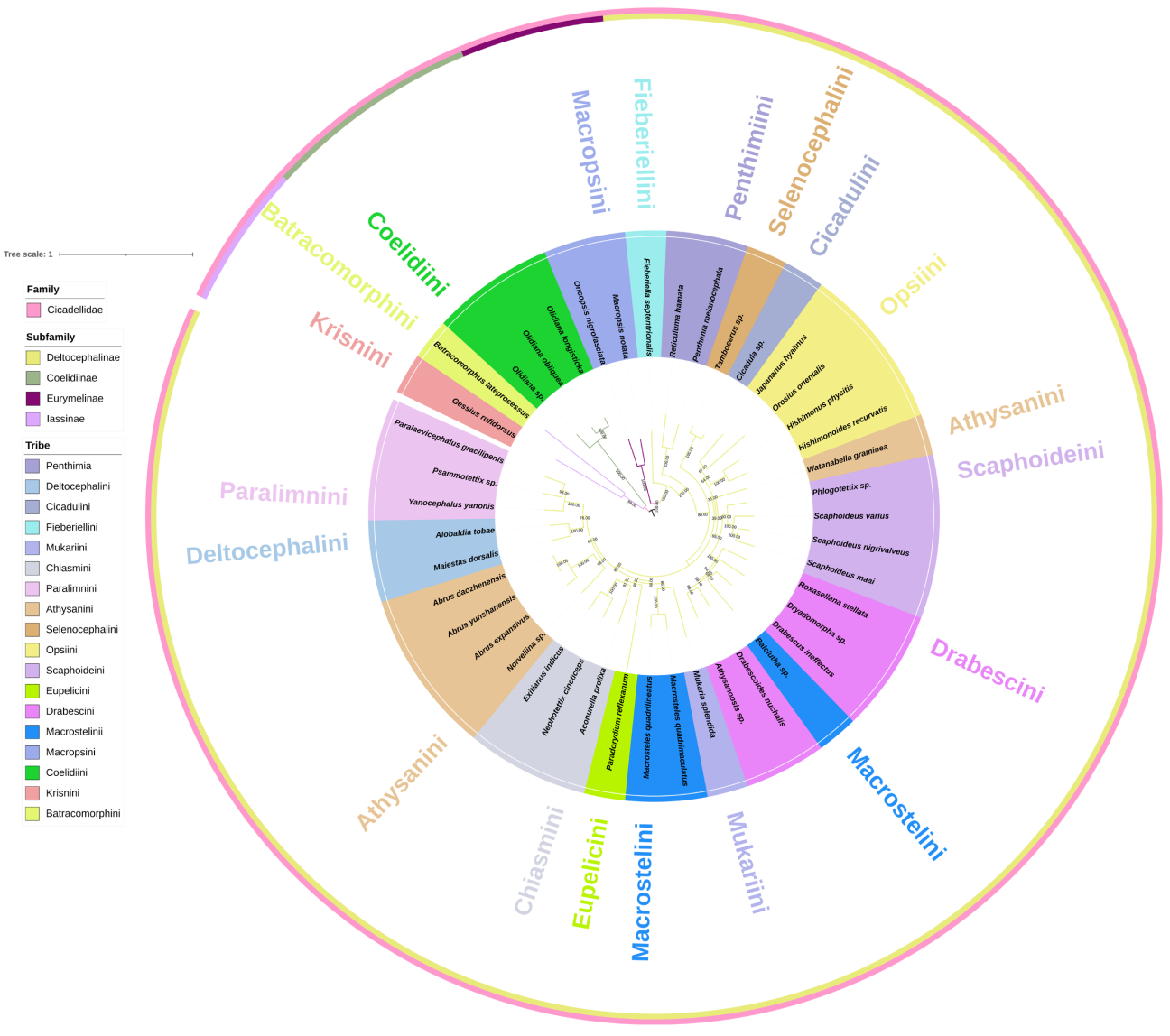


**Supplementary Figure S13.** The phylogenetic tree produced by maximum likelihood analyses based on PCG123_AA datasets. Numbers at nodes are bootstrap support values (BS).


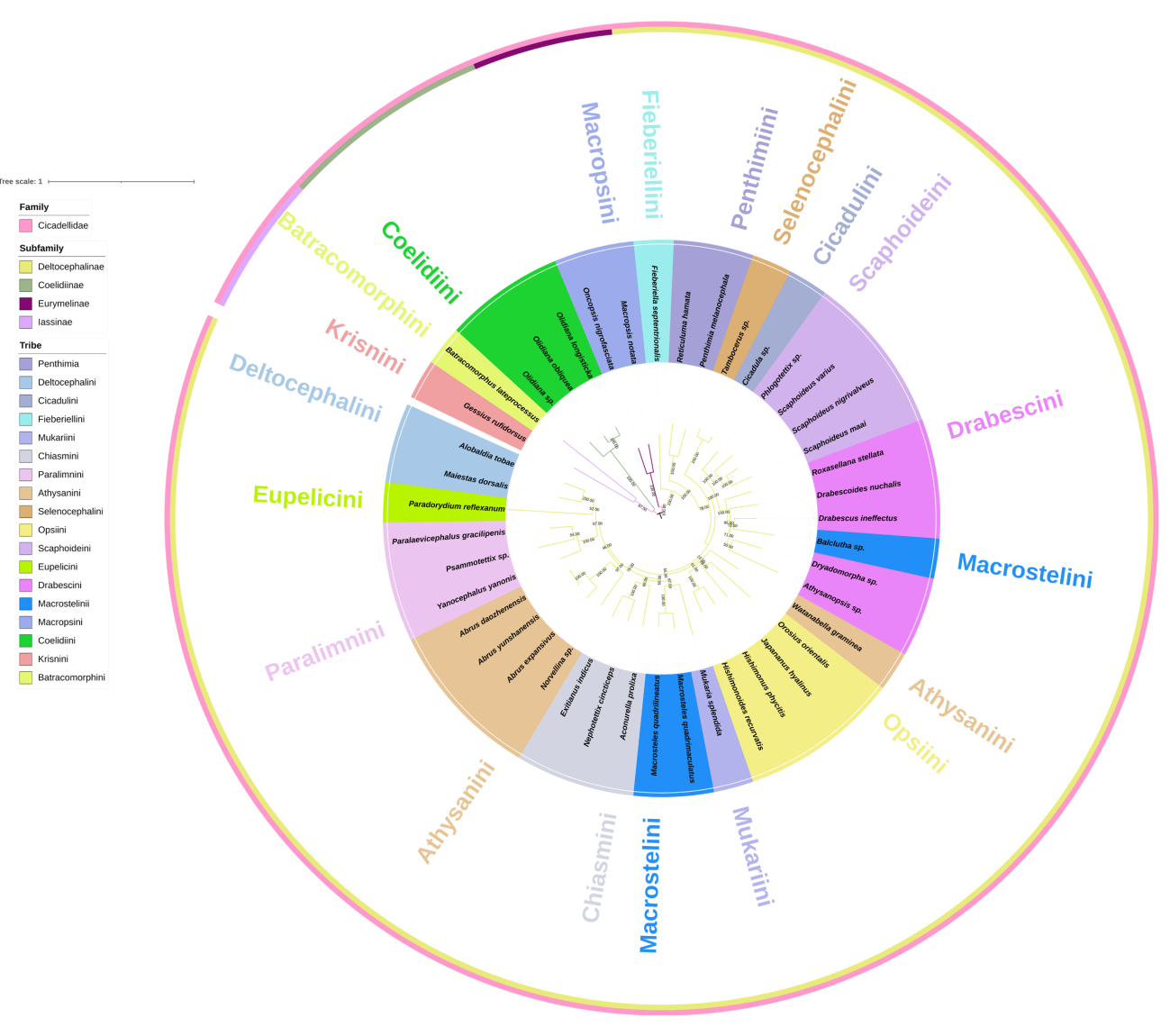


**Supplementary Figure S14.** The phylogenetic tree produced by maximum likelihood analyses based on PCG123_AA+ 2 rRNA datasets. Numbers at nodes are bootstrap support values (BS).


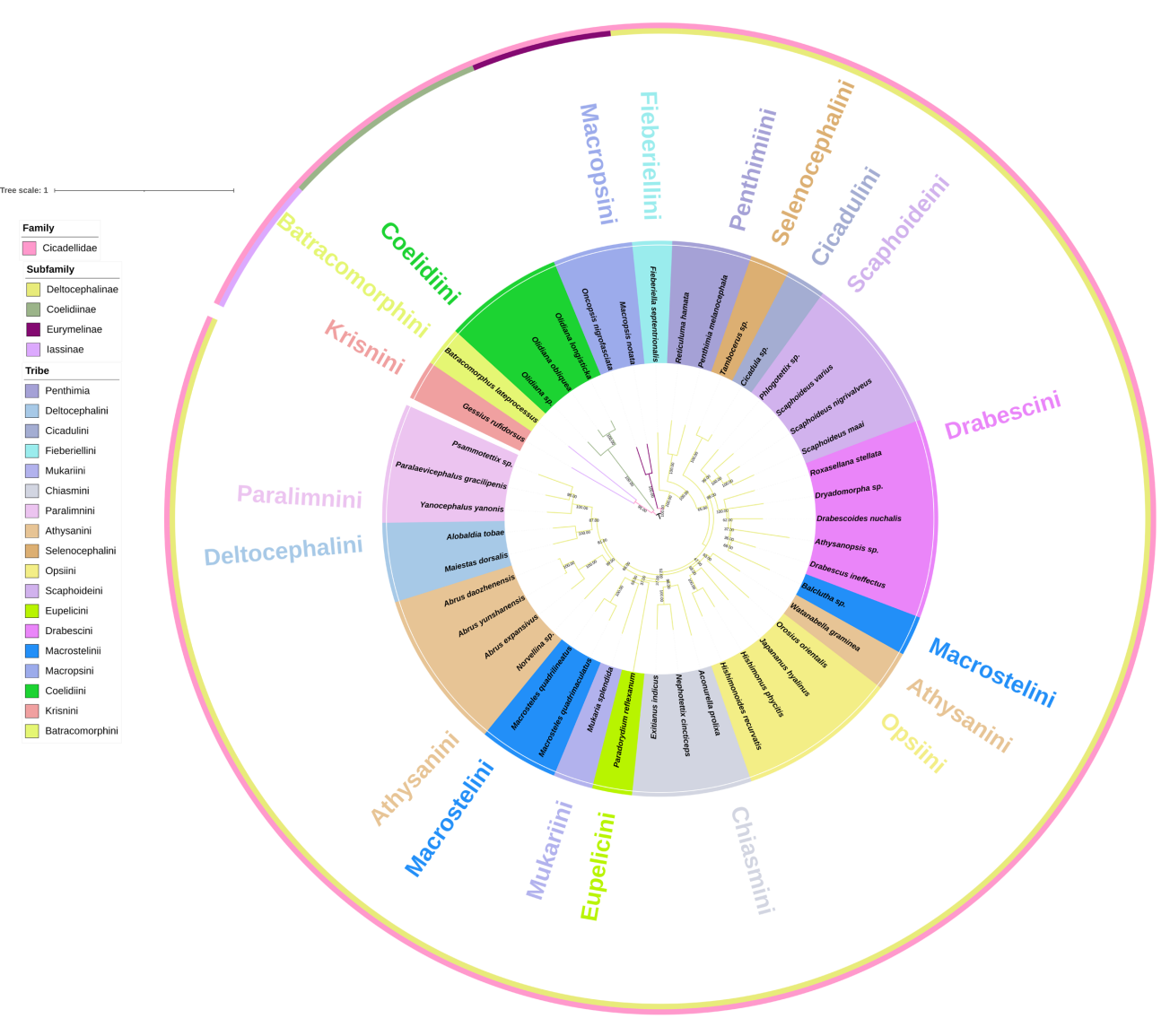


**Supplementary Figure S15.** The phylogenetic tree produced by maximum likelihood analyses based on PCG123_AA+ 2 rRNA + 22 tRNA datasets. Numbers at nodes are bootstrap support values (BS).
