# Supplementary material for: Comparative mitochondrial genome analysis of three leafhopper species of the genus Abrus Dai & Zhang (Hemiptera: Cicadellidae: Deltocephalinae) from China with phylogenetic implication
Source: BMC Genomics. 2023 Nov 27;24:714. doi: 10.1186/s12864-023-09809-0 (PMC10680345; doi:10.1186/s12864-023-09809-0)
Supplement: Supplementary file 2 — Additional file 2: Supplementary Table S1. Organization of the Abrus daozhenensis mitochondrial genome. Supplementary Table S2. Organization of the Abrus yunshanensis mitochondrial genome. Supplementary Table S3. Organization of the Abrus expansivus mitochondrial genome. Supplementary Table S4. Overall A+T content in the first (P1), second (P2) and third (P3) codon positions. Supplementary Table S5. The best partitioning schemes and models for the Bayesian inference (BI) method based on 123PCG dataset selected by PartitionFinder. Supplementary Table S6. The best partitioning schemes and models for the Bayesian inference (BI) method based on 123PCG + 2 rRNA dataset selected by PartitionFinder. Supplementary Table S7. The best partitioning schemes and models for the Bayesian inference (BI) method based on 123PCG + 2 rRNA + 22 tRNA dataset selected by PartitionFinder. Supplementary Table S8. The best partitioning schemes and models for the Bayesian inference (BI) method based on 123PCG_AA dataset selected by PartitionFinder. Supplementary Table S9. The best partitioning schemes and models for the Bayesian inference (BI) method based on 123PCG_AA + 2 rRNA dataset selected by PartitionFinder. Supplementary Table S10. The best partitioning schemes and models for the Bayesian inference (BI) method based on 123PCG_AA + 2 rRNA + 22 tRNA dataset selected by PartitionFinder. Supplementary Table S11. The best partitioning schemes and models for maximum-likelihood (ML) analyses on 123PCG dataset selected by PartitionFinder. Supplementary Table S12. The best partitioning schemes and models for maximum-likelihood (ML) analyses on 123PCG + 2rRNA dataset selected by PartitionFinder. Supplementary Table S13. The best partitioning schemes and models for maximum-likelihood (ML) analyses on 123PCG + 2rRNA + 22tRNA dataset selected by PartitionFinder. Supplementary Table S14. The best partitioning schemes and models for maximum-likelihood (ML) analyses on 123PCG_AA dataset selected by Pa [file 12864_2023_9809_MOESM2_ESM.docx]

**Supplementary Table S1.** Organization of the *Abrus daozhenensis* mitochondrial genome.

| NO. | Gene | Direction | Location | Length | Start codon | Stop codon | Anticodon | Intergenic nucleotides |
| --- | --- | --- | --- | --- | --- | --- | --- | --- |
|  |  |  |  |  |  |  |  |  |
| 1 | tRNA-Ile | J | 1~65 | 65 |  |  | GAT | -3 |
| 2 | tRNA-Gln | N | 63~131 | 69 |  |  | TTG | 0 |
| 3 | tRNA-Met | J | 132~197 | 66 |  |  | CAT | 0 |
| 4 | nad2 | J | 198~1172 | 975 | ATT | TAG |  | 0 |
| 5 | tRNA-Trp | J | 1173~1242 | 70 |  |  | TCA | -8 |
| 6 | tRNA-Cys | N | 1235~1296 | 62 |  |  | GCA | 2 |
| 7 | tRNA-Tyr | N | 1299~1364 | 66 |  |  | GTA | 38 |
| 8 | cox1 | J | 1403~2941 | 1539 | ATG | TAA |  | 0 |
| 9 | tRNA-Leu | J | 2942~3006 | 65 |  |  | TAA | 0 |
| 10 | cox2 | J | 3007~3685 | 679 | ATA | T |  | 0 |
| 11 | tRNA-Lys | J | 3686~3755 | 70 |  |  | CTT | 0 |
| 12 | tRNA-Asp | J | 3756~3823 | 68 |  |  | GTC | 0 |
| 13 | atp8 | J | 3824~3976 | 153 | ATA | TAA |  | -7 |
| 14 | atp6 | J | 3970~4623 | 654 | ATG | TAA |  | 5 |
| 15 | cox3 | J | 4629~5408 | 780 | ATG | TAA |  | 5 |
| 16 | tRNA-Gly | J | 5414~5473 | 60 |  |  | TCC | 0 |
| 17 | nad3 | J | 5474~5827 | 354 | ATT | TAG |  | -2 |
| 18 | tRNA-Ala | J | 5826~5886 | 61 |  |  | TGC | 0 |
| 19 | tRNA-Arg | J | 5887~5951 | 65 |  |  | TCG | -1 |
| 20 | tRNA-Asn | J | 5951~6024 | 74 |  |  | GTT | -1 |
| 21 | tRNA-Ser | J | 6024~6089 | 66 |  |  | GCT | -4 |
| 22 | tRNA-Glu | J | 6086~6151 | 66 |  |  | TTC | 0 |
| 23 | tRNA-Phe | N | 6152~6214 | 63 |  |  | GAA | 3 |
| 24 | nad5 | N | 6218~7891 | 1674 | ATT | TAG |  | 0 |
| 25 | tRNA-His | N | 7892~7952 | 61 |  |  | GTG | -1 |
| 26 | nad4 | N | 7952~9259 | 1308 | ATG | TAA |  | -7 |
| 27 | nad4l | N | 9253~9528 | 276 | ATT | TAA |  | 2 |
| 28 | tRNA-Thr | J | 9531~9593 | 63 |  |  | TGT | 0 |
| 29 | tRNA-Pro | N | 9594~9659 | 66 |  |  | TGG | 2 |
| 30 | nad6 | J | 9662~10144 | 483 | ATT | TAA |  | -1 |
| 31 | cob | J | 10144~11280 | 1137 | ATG | TAA |  | 0 |
| 32 | tRNA-Ser | J | 11281~11342 | 62 |  |  | TGA | -1 |
| 33 | nad1 | N | 11342~12274 | 933 | ATA | TAA |  | 0 |
| 34 | tRNA-Leu | N | 12275~12339 | 65 |  |  | TAG | 0 |
| 35 | 16S-rRNA | N | 12340~13543 | 1204 |  |  |  | 0 |
| 36 | tRNA-Val | N | 13544~13611 | 68 |  |  | TAC | 0 |
| 37 | 12S-rRNA | N | 13612~14356 | 745 |  |  |  | 0 |
| 38 | CR |  | 14357~16391 | 2035 |  |  |  | 0 |

**Supplementary Table S2.** Organization of the *Abrus yunshanensis* mitochondrial genome.

| NO. | Gene | Direction | Location | Length | Start codon | Stop codon | Anticodon | Intergenic nucleotides |
| --- | --- | --- | --- | --- | --- | --- | --- | --- |
|  |  |  |  |  |  |  |  |  |
|  |  |  |  |  |  |  |  |  |
| 1 | tRNA-Ile | J | 1~65 | 65 |  |  | GAT | -3 |
| 2 | tRNA-Gln | N | 63~131 | 69 |  |  | TTG | 0 |
| 3 | tRNA-Met | J | 132~197 | 66 |  |  | CAT | 0 |
| 4 | nad2 | J | 198~1172 | 975 | ATT | TAG |  | 0 |
| 5 | tRNA-Trp | J | 1173~1242 | 70 |  |  | TCA | -8 |
| 6 | tRNA-Cys | N | 1235~1296 | 62 |  |  | GCA | 2 |
| 7 | tRNA-Tyr | N | 1299~1364 | 66 |  |  | GTA | 39 |
| 8 | cox1 | J | 1404~2942 | 1539 | ATG | TAA |  | 1 |
| 9 | tRNA-Leu | J | 2944~3008 | 65 |  |  | TAA | 0 |
| 10 | cox2 | J | 3009~3687 | 679 | ATA | T |  | 0 |
| 11 | tRNA-Lys | J | 3688~3757 | 70 |  |  | CTT | 0 |
| 12 | tRNA-Asp | J | 3758~3823 | 66 |  |  | GTC | 0 |
| 13 | atp8 | J | 3824~3976 | 153 | ATA | TAA |  | -7 |
| 14 | atp6 | J | 3970~4623 | 654 | ATG | TAA |  | 5 |
| 15 | cox3 | J | 4629~5408 | 780 | ATG | TAG |  | 5 |
| 16 | tRNA-Gly | J | 5414~5475 | 62 |  |  | TCC | 0 |
| 17 | nad3 | J | 5476~5829 | 354 | ATT | TAG |  | -2 |
| 18 | tRNA-Ala | J | 5828~5888 | 61 |  |  | TGC | 0 |
| 19 | tRNA-Arg | J | 5889~5953 | 65 |  |  | TCG | -1 |
| 20 | tRNA-Asn | J | 5953~6023 | 71 |  |  | GTT | -1 |
| 21 | tRNA-Ser | J | 6023~6088 | 66 |  |  | GCT | -4 |
| 22 | tRNA-Glu | J | 6085~6150 | 66 |  |  | TTC | 0 |
| 23 | tRNA-Phe | N | 6151~6213 | 63 |  |  | GAA | 3 |
| 24 | nad5 | N | 6217~7890 | 1674 | ATT | TAG |  | 0 |
| 25 | tRNA-His | N | 7891~7951 | 61 |  |  | GTG | -1 |
| 26 | nad4 | N | 7951~9258 | 1308 | ATG | TAA |  | -7 |
| 27 | nad4l | N | 9252~9527 | 276 | ATT | TAA |  | 2 |
| 28 | tRNA-Thr | J | 9530~9592 | 63 |  |  | TGT | 0 |
| 29 | tRNA-Pro | N | 9593~9658 | 66 |  |  | TGG | 2 |
| 30 | nad6 | J | 9661~10143 | 483 | ATT | TAA |  | -1 |
| 31 | cob | J | 10143~11279 | 1137 | ATG | TAA |  | 0 |
| 32 | tRNA-Ser | J | 11280~11341 | 62 |  |  | TGA | -1 |
| 33 | nad1 | N | 11341~12273 | 933 | ATA | TAA |  | 0 |
| 34 | tRNA-Leu | N | 12274~12338 | 65 |  |  | TAG | 0 |
| 35 | 16S-rRNA | N | 12339~13541 | 1203 |  |  |  | 0 |
| 36 | tRNA-Val | N | 13542~13608 | 67 |  |  | TAC | 0 |
| 37 | 12S-rRNA | N | 13609~14351 | 743 |  |  |  | 0 |
| 38 | CR |  | 14352~16298 | 1947 |  |  |  | 0 |

**Supplementary Table S3.** Organization of the *Abrus expansivus* mitochondrial genome.

| NO. | Gene | Direction | Location | Length | Start codon | Stop codon | Anticodon | Intergenic nucleotides |
| --- | --- | --- | --- | --- | --- | --- | --- | --- |
|  |  |  |  |  |  |  |  |  |
| 1 | tRNA-Ile | J | 1~68 | 68 |  |  | GAT | 0 |
| 2 | tRNA-Gln | N | 69~137 | 69 |  |  | TTG | -1 |
| 3 | tRNA-Met | J | 137~202 | 66 |  |  | CAT | 0 |
| 4 | nad2 | J | 203~1177 | 975 | ATT | TAG |  | 0 |
| 5 | tRNA-Trp | J | 1178~1253 | 76 |  |  | TCA | -8 |
| 6 | tRNA-Cys | N | 1246~1306 | 61 |  |  | GCA | 5 |
| 7 | tRNA-Tyr | N | 1312~1378 | 67 |  |  | GTA | 18 |
| 8 | cox1 | J | 1397~2935 | 1,539 | ATA | TAA |  | 0 |
| 9 | tRNA-Leu | J | 2936~2998 | 65 |  |  | TAA | 1 |
| 10 | cox2 | J | 3000~3678 | 679 | ATA | T |  | 0 |
| 11 | tRNA-Lys | J | 3679~3750 | 72 |  |  | CTT | 0 |
| 12 | tRNA-Asp | J | 3751~3820 | 70 |  |  | GTC | 0 |
| 13 | atp8 | J | 3821~3973 | 153 | ATA | TAA |  | -7 |
| 14 | atp6 | J | 3967~4620 | 654 | ATG | TAA |  | -1 |
| 15 | cox3 | J | 4620~5399 | 780 | ATG | TAA |  | 5 |
| 16 | tRNA-Gly | J | 5405~5465 | 61 |  |  | TCC | 0 |
| 17 | nad3 | J | 5466~5819 | 354 | ATT | TAA |  | -1 |
| 18 | tRNA-Ala | J | 5819~5879 | 61 |  |  | TGC | 0 |
| 19 | tRNA-Arg | J | 5880~5944 | 65 |  |  | TCG | -1 |
| 20 | tRNA-Asn | J | 5944~6015 | 72 |  |  | GTT | -1 |
| 21 | tRNA-Ser | J | 6015~6080 | 66 |  |  | GCT | -3 |
| 22 | tRNA-Glu | J | 6078~6138 | 61 |  |  | TTC | -1 |
| 23 | tRNA-Phe | N | 6138~6199 | 62 |  |  | GAA | 6 |
| 24 | nad5 | N | 6206~7879 | 1,674 | ATT | TAG |  | 0 |
| 25 | tRNA-His | N | 7880~7941 | 62 |  |  | GTG | 2 |
| 26 | nad4 | N | 7944~9248 | 1,305 | ATG | TAA |  | -4 |
| 27 | nad4l | N | 9245~9520 | 276 | ATT | TAG |  | 2 |
| 28 | tRNA-Thr | J | 9523~9585 | 63 |  |  | TGT | 0 |
| 29 | tRNA-Pro | N | 9586~9651 | 66 |  |  | TGG | 8 |
| 30 | nad6 | J | 9660~10136 | 477 | ATA | TAA |  | -1 |
| 31 | cob | J | 10136~11272 | 1,137 | ATG | TAA |  | 0 |
| 32 | tRNA-Ser | J | 11273~11336 | 64 |  |  | TGA | -1 |
| 33 | nad1 | N | 11336~12268 | 933 | ATA | TAA |  | 0 |
| 34 | tRNA-Leu | N | 12269~12334 | 66 |  |  | TAG | 0 |
| 35 | 16S-rRNA | N | 12335~13538 | 1,204 |  |  |  | 0 |
| 36 | tRNA-Val | N | 13539~13603 | 65 |  |  | TAC | 0 |
| 37 | 12S-rRNA | N | 13604~14359 | 756 |  |  |  | 0 |
| 38 | CR |  | 14358~15904 | 1545 |  |  |  | 0 |

**Supplementary Table S4.** Overall A+T content in the first (P1), second (P2) and third (P3) codon positions.

| Species | | P1 | P2 | P3 |  |
| --- | --- | --- | --- | --- | --- |
| *A. daozhenensis* | | 80.1 | 70.2 | 73.4 |  |
| *A. yunshanrnsis* | | 80.4 | 70.2 | 73.4 |  |
| *A. expansivus* | | 73.7 | 73.0 | 72.0 |  |
| Mean | | 78.1 | 71.1 | 72.9 |  |
|  |  |  |  |  |  |

**Supplementary Table S5.** The best partitioning schemes and models for the Bayesian inference (BI) method based on 123PCG dataset selected by PartitionFinder.

| **Dataset** | **Partitions** | **Partitioning schemes** | **Sites** | **Best model** |
| --- | --- | --- | --- | --- |
| **123PCG** | P1 | cytb_pos1, cox3_pos1, cox2_pos1 | 859 | GTR+I+G |
|  | P2 | cox1_pos2, cox3_pos2, cox2_pos2, cytb_pos2 | 1366 | GTR+I+G |
|  | P3 | nad6_pos3, nad3_pos3, atp8_pos3, atp6_pos3, cytb_pos3, cox1_pos3, cox2_pos3, cox3_pos3 | 1885 | GTR+I+G |
|  | P4 | atp6_pos1, nad6_pos1 | 354 | GTR+I+G |
|  | P5 | nad2_pos2, nad3_pos2, atp8_pos2, atp6_pos2, nad6_pos2 | 829 | GTR+I+G |
|  | P6 | atp8_pos1, nad2_pos1, nad3_pos1 | 475 | GTR+I+G |
|  | P7 | cox1_pos1 | 507 | GTR+I+G |
|  | P8 | nad5_pos1, nad4L_pos1, nad4_pos1, nad1_pos1 | 1362 | GTR+I+G |
|  | P9 | nad1_pos2, nad5_pos2, nad4L_pos2, nad4_pos2 | 1362 | GTR+I+G |
|  | P10 | nad1_pos3, nad4L_pos3, nad4_pos3, nad5_pos3 | 1362 | GTR+I+G |
|  | P11 | nad2_pos3 | 310 | GTR+I+G |
|  |  |  |  |  |

**Supplementary Table S6.** The best partitioning schemes and models for the Bayesian inference (BI) method based on 123PCG + 2 rRNA dataset selected by PartitionFinder.

| **Dataset** | | **Partitions** | | **Partitioning schemes** | **Sites** | | **Best model** | |
| --- | --- | --- | --- | --- | --- | --- | --- | --- |
| **123PCG + 2 rRNA** | P1 | | cytb_pos1, cox3_pos1, cox2_pos1 | | 859 | | GTR+I+G | |
|  | P2 | | cox1_pos2, cox3_pos2, cox2_pos2, cytb_pos2 | | 1366 | | GTR+I+G | |
|  | P3 | | atp8_pos3, atp6_pos3, nad6_pos3, nad3_pos3, cytb_pos3, cox1_pos3, cox2_pos3, cox3_pos3 | | 1885 | | GTR+I+G | |
|  | P4 | | atp6_pos1, nad6_pos1 | | 354 | | GTR+I+G | |
|  | P5 | | nad3_pos2, nad2_pos2, atp8_pos2, nad6_pos2, atp6_pos2 | | 829 | | GTR+I+G | |
|  | P6 | | atp8_pos1, nad2_pos1, nad3_pos1 | | 475 | | GTR+I+G | |
|  | P7 | | cox1_pos1 | | 507 | | GTR+I+G | |
|  | P8 | | nad5_pos1, nad4L_pos1, nad1_pos1, nad4_pos1 | | 1362 | | GTR+I+G | |
|  | P9 | | nad1_pos22, nad5_pos2, nad4_pos2, nad4L_pos2 | | 1362 | | GTR+I+G | |
|  | P10 | | nad1_pos3, nad4L_pos3, nad4_pos3, nad5_pos3 | | 1362 | | GTR+I+G | |
|  | P11 | | P11: (nad2_mafft_NT_removed_chars_gb_codon3) | | 310 | | GTR+I+G | |
|  | P12 | | rrnL | | 1126 | | GTR+G | |
|  | P13 | | rrnS | | 724 | | GTR+I+G | |
|  | |  | |  | |  | |  |

**Supplementary Table S7.** The best partitioning schemes and models for the Bayesian inference (BI) method based on 123PCG + 2 rRNA + 22 tRNA dataset selected by PartitionFinder.

| **Dataset** | **Partitions** | **Partitioning schemes** | **Sites** | **Best model** |
| --- | --- | --- | --- | --- |
| **123PCG + 2 rRNA + 22 tRNA** | P1 | cox1_pos1, cytb_pos1 | 884 | GTR+I+G |
|  | P2 | cox1_pos2, cox3_pos2, cox2_pos2, cytb_pos2 | 1366 | GTR+I+G |
|  | P3 | nad6_pos3, nad3_pos3, atp8_pos3, atp6_pos3, cytb_pos3, cox1_pos3, cox2_pos3, cox3_pos3 | 1885 | GTR+I+G |
|  | P4 | atp6_pos1, nad6_pos1 | 354 | GTR+I+G |
|  | P5 | nad3_pos2, nad2_pos2, atp8_pos2, atp6_pos2, nad6_pos2 | 829 | GTR+I+G |
|  | P6 | atp8_pos1, nad2_pos1, nad3_pos1 | 475 | GTR+I+G |
|  | P7 | cox2_pos1, trnM, cox3_pos1, trnK | 617 | GTR+I+G |
|  | P8 | trnV, nad1_pos1, trnA, trnS1, trnP, trnF | 609 | GTR+I+G |
|  | P9 | nad1_pos2, nad5_pos2, nad4L_pos2, nad4_pos2 | 1362 | GTR+I+G |
|  | P10 | nad4_pos3, nad5_pos3, nad1_pos3, nad4L_pos3 | 1362 | GTR+I+G |
|  | P11 | nad2_pos3 | 310 | GTR+I+G |
|  | P12 | nad5_pos1, nad4L_pos1, nad4_pos1, trnL1, trnQ | 1188 | GTR+I+G |
|  | P13 | rrnL | 1126 | GTR+G |
|  | P14 | rrnS | 724 | GTR+I+G |
|  | P15 | trnY, trnH, trnT, trnC, trnL2, trnS2, trnI, trnD, trnW | 569 | GTR+G |
|  | P16 | trnN, trnE, trnG, trnR | 244 | GTR+G |
|  |  |  |  |  |

**Supplementary Table S8.** The best partitioning schemes and models for the Bayesian inference (BI) method based on 123PCG_AA dataset selected by PartitionFinder.

| **Dataset** | **Partitions** | **Partitioning schemes** | **Sites** | **Best model** |
| --- | --- | --- | --- | --- |
| **123PCG_AA** | P1 | cytb | 378 | LG+I+G |
|  | P2 | atp6l, cox3, cox2, nad1 | 1011 | LG+I+G |
|  | P3 | atp8 | 49 | LG+I+G |
|  | P4 | cox1 | 516 | LG+I+G |
|  | P5 | nad2, nad6, nad4L | 513 | LG+I+G |
|  | P6 | nad3, nad5, nad4 | 1105 | LG+I+G |
|  |  |  |  |  |

**Supplementary Table S9.** The best partitioning schemes and models for the Bayesian inference (BI) method based on 123PCG_AA + 2 rRNA dataset selected by PartitionFinder.

| **Dataset** | | **Partitions** | **Partitioning scheme** | **Sites** | **Best model** |
| --- | --- | --- | --- | --- | --- |
| **123PCG_AA + 2 rRNA** | P1 | | cytb, cox2, cox3, nad1 | 1190 | LG+I+G |
|  | P2 | | atp6, nad3, nad4L | 438 | LG+I+G |
|  | P3 | | atp8 | 53 | LG+I+G |
|  | P4 | | cox1 | 518 | LG+I+G |
|  | P5 | | nad6, nad2 | 528 | LG+I+G |
|  | P6 | | nad4, nad5 | 1016 | LG+I+G |
|  | P7 | | rrnL | 1126 | LG+G |
|  | P8 | | rrnS | 724 | LG+I+G |
|  | |  |  |  |  |

**Supplementary Table S10.** The best partitioning schemes and models for the Bayesian inference (BI) method based on 123PCG_AA + 2 rRNA + 22 tRNA dataset selected by PartitionFinder.

| **Dataset** | **Partitions** | **Partitioning scheme** | **Sites** | **Best model** |
| --- | --- | --- | --- | --- |
| **123PCG_AA + 2 rRNA + 22 tRNA** | P1 | cytb | 378 | LG+I+G |
|  | P2 | atp6, cox3, nad1, cox2 | 1011 | LG+I+G |
|  | P3 | atp8 | 49 | LG+I+G |
|  | P4 | trnK, cox1, trnI, trnM | 713 | LG+I+G |
|  | P5 | nad2, nad3, trnDl, nad6, nad4L | 693 | LG+I+G |
|  | P6 | nad4, nad5 | 988 | LG+I+G |
|  | P7 | trnF, trnS1, trnH, trnQ, rrnL, trnL1 | 1441 | LG+G |
|  | P8 | trnE, rrnS | 784 | LG+I+G |
|  | P9 | trnL2, trnNl, trnR, trnY, trnV, trnA, trnG, trnP, trnC | 552 | LG+G |
|  | P10 | trnT, trnW, trnS2 | 196 | LG+G |
|  |  |  |  |  |

**Supplementary Table S11.** The best partitioning schemes and models for maximum-likelihood (ML) analyses on 123PCG dataset selected by PartitionFinder.

| **Dataset** | **Partitions** | **Partitioning scheme** | **Best model** |
| --- | --- | --- | --- |
| **PCG123** | P1 | cytb_pos1, cox2_pos1, cox3_pos1 | GTR+F+I+G4 |
|  | P2 | cytb_pos2, cox1_pos2, cox2_pos2, cox3_pos2 | TVM+F+R3 |
|  | P3 | cytb_pos3 | TPM2u+F+I+I+R4 |
|  | P4 | atp6_pos1, atp8_pos2, nad6_pos1 | GTR+F+I+G4 |
|  | P5 | atp6_pos2, nad2_pos2, nad3_pos2, nad6_pos2 | TVM+F+I+I+R4 |
|  | P6 | atp6_pos3, atp8_pos3, cox2_pos3, nad3_pos3, nad6_pos3 | TPM3u+F+I+I+R3 |
|  | P7 | atp8_pos1, nad2_pos1, nad3_pos1 | TIM+F+R4 |
|  | P8 | cox1_pos1 | GTR+F+I+G4 |
|  | P9 | cox1_pos3, cox3_pos3 | HKY+F+I+I+R4 |
|  | P10 | nad1_pos1 | GTR+F+I+G4 |
|  | P11 | nad1_pos2 | GTR+F+I+G4 |
|  | P12 | nad1_pos3, nad4L_pos3 | HKY+F+R5 |
|  | P13 | nad2_pos3 | GTR+F+I+G4 |
|  | P14 | nad4L_pos1, nad4_pos1, nad5_pos1 | GTR+F+R4 |
|  | P15 | nad4L_pos2, nad4_pos2, nad5_pos2 | GTR+F+I+G4 |
|  | P16 | nad4_pos3, nad5_pos3 | TPM3u+F+R5 |
|  |  |  |  |

**Supplementary Table S12.** The best partitioning schemes and models for maximum-likelihood (ML) analyses on 123PCG + 2rRNA dataset selected by PartitionFinder.

| **Dataset** | **Partitions** | **Partitioning scheme** | **Best model** |
| --- | --- | --- | --- |
| **123PCG + 2rRNA** | P1 | cytb_pos1, cox2_pos1, cox3_pos1 | GTR+F+I+I+R4 |
|  | P2 | cytb_pos2, cox1_pos2, cox2_pos2, cox3_pos2 | TVM+F+R3 |
|  | P3 | cytb_pos3 | TPM3u+F+I+I+R4 |
|  | P4 | atp6_pos1, atp8_pos2, nad6_pos1 | GTR+F+I+G4 |
|  | P5 | atp6_pos2, nad2_pos2, nad3_pos2, nad6_pos2 | TVM+F+I+I+R4 |
|  | P6 | atp6_pos3, atp8_pos3, nad3_pos3 | TPM3u+F+R3 |
|  | P7 | atp8_pos1, nad2_pos1, nad3_pos1 | TIM+F+R4 |
|  | P8 | cox1_pos1 | GTR+F+I+G4 |
|  | P9 | cox1_pos3, cox3_pos3 | HKY+F+I+I+R4 |
|  | P10 | cox2_pos3, nad6_pos3 | HKY+F+G4 |
|  | P11 | nad1_pos1 | GTR+F+I+G4 |
|  | P12 | nad1_pos2 | GTR+F+I+G4 |
|  | P13 | nad1_pos3, nad4L_pos3, nad4_pos3 | TIM3+F+R5 |
|  | P14 | nad2_pos3 | GTR+F+I+G4 |
|  | P15 | nad4L_pos1, nad4_pos1, nad5_pos1 | GTR+F+R5 |
|  | P16 | nad4L_pos2, nad4_pos2, nad5_pos2 | GTR+F+I+G4 |
|  | P17 | nad5_pos3 | GTR+F+I+G4 |
|  | P18 | rrnL, rrnS | GTR+F+I+I+R4 |
|  |  |  |  |

**Supplementary Table S13.** The best partitioning schemes and models for maximum-likelihood (ML) analyses on 123PCG + 2rRNA + 22tRNA dataset selected by PartitionFinder.

| **Dataset** | **Partitions** | **Partitioning scheme** | **Best model** |
| --- | --- | --- | --- |
| **123PCG + 2rRNA + 22tRNA** | P1 | cytb_pos1, cox2_pos1, cox2_pos2, cox2_pos3, cox3_pos1, nad3_pos1, trnK, trnM | GTR+F+I+I+R4 |
|  | P2 | atp6_pos1, nad3_pos3, nad6_pos1 | TIM+F+I+G4 |
|  | P3 | atp8_pos1, nad4L_pos3, rrnL, trnG, trnL1, trnT, trnV | TVM+F+R4 |
|  | P4 | atp8_pos2, trnD, trnH, trnW | TIM3+F+G4 |
|  | P5 | atp8_pos3, nad3_pos2 | TIM2+F+I+G4 |
|  | P6 | cox1_pos1, cox1_pos2, cox1_pos3 | GTR+F+I+I+R4 |
|  | P7 | nad1_pos1, nad1_pos2, nad1_pos3, nad4L_pos1 | TVM+F+I+G4 |
|  | P8 | nad2_pos1, nad4L_pos2 | GTR+F+I+G4 |
|  | P9 | nad4_pos1, nad4_pos2, nad4_pos3, nad5_pos1, nad5_pos2, nad5_pos3 | TVM+F+I+G4 |
|  | P10 | rrnS, trnN | GTR+F+I+G4 |
|  | P11 | trnA, trnI, trnP | TIM3+F+R4 |
|  | P12 | trnC, trnE, trnL2, trnR, trnS2 | TPM3u+F+R4 |
|  | P13 | trnF, trnQ, trnS1 | GTR+F+G4 |
|  | P14 | trnY | GTR+F+I+G4 |
|  |  |  |  |

**Supplementary Table S14.** The best partitioning schemes and models for maximum-likelihood (ML) analyses on 123PCG_AA dataset selected by PartitionFinder.

| **Dataset** | **Partitions** | **Partitioning scheme** | **Best model** |
| --- | --- | --- | --- |
| **123PCG_AA** | P1 | cytb_pos1, cytb_pos2, cytb_pos3, cox2_pos1, cox2_pos2, cox2_pos3, cox3_pos1 | mtART+F+R5 |
|  | P2 | atp6_pos1, nad2_pos2, nad3_pos1, nad3_pos2, nad4L_pos2, nad6_pos2, nad6_pos3 | mtZOA+F+R5 |
|  | P3 | atp8_pos1 | LG+F+I+G4 |
|  | P4 | cox1_pos1, cox1_pos2, cox1_pos3 | mtART+R4 |
|  | P5 | nad1_pos1 | LG+F+I+G4 |
|  | P6 | nad2_pos1, nad2_pos3, nad3_pos3, nad4L_pos1 | mtZOA+F+R5 |
|  | P7 | nad4L_pos3, nad4_pos1, nad6_pos1 | mtZOA+F+I+I+R5 |
|  | P8 | nad5_pos1 | LG+F+I+G4 |
|  |  |  |  |

**Supplementary Table S15.** The best partitioning schemes and models for maximum-likelihood (ML) analyses on 123PCG_AA + 2rRNA dataset selected by PartitionFinder.

| **Dataset** | **Partitions** | **Partitioning scheme** | **Best model** |
| --- | --- | --- | --- |
| **123PCG_AA + 2rRNA** | P1 | cytb_pos1, cytb_pos2, cytb_pos3, cox2_pos3 | mtART+F+I+G4 |
|  | P2 | atp6_pos1, nad2_pos2, nad3_pos1, nad3_pos2, nad6_pos2, nad6_pos3 | mtZOA+F+I+G4 |
|  | P3 | atp8_pos1 | LG+F+I+G4 |
|  | P4 | cox1_pos1, cox1_pos2, cox1_pos3 | mtART+R4 |
|  | P5 | cox2_pos1, cox2_pos2, cox3_pos1 | mtART+F+I+G4 |
|  | P6 | nad1_pos1 | LG+F+I+G4 |
|  | P7 | nad2_pos1, nad2_pos3, nad3_pos3, nad4L_pos1, nad6_pos1 | mtZOA+F+R5 |
|  | P8 | nad4L_pos2 | LG+F+I+G4 |
|  | P9 | nad4L_pos3, nad5_pos1 | mtZOA+F+R5 |
|  | P10 | nad4_pos1 | LG+F+I+G4 |
|  | P11 | rrnL, rrnS | Blosum62+F+I+I+R4 |
|  |  |  |  |

**Supplementary Table S16.** The best partitioning schemes and models for maximum-likelihood (ML) analyses on 123PCG_AA + 2rRNA + 22tRNA dataset selected by PartitionFinder.

| **Dataset** | **Partitions** | **Partitioning scheme** | **Best model** |
| --- | --- | --- | --- |
| **123PCG_AA + 2rRNA + 22tRNA** | P1 | cytb_pos1, cytb_pos2 | mtART+R4 |
|  | P2 | cytb_pos3, cox2_pos1 | mtART+F+I+G4 |
|  | P3 | atp6_pos1, nad2_pos2, nad3_pos1, nad3_pos2, nad4L_pos2, nad6_pos2, nad6_pos3 | mtZOA+F+R5 |
|  | P4 | atp8_pos1 | LG+F+I+G4 |
|  | P5 | cox1_pos1, cox1_pos2, cox1_pos3 | mtART+R4 |
|  | P6 | cox2_pos2, cox2_pos3, cox3_pos1 | mtART+F+I+G4 |
|  | P7 | nad1_pos1 | LG+F+I+G4 |
|  | P8 | nad2_pos1, nad2_pos3, nad3_pos3, nad4L_pos1, nad4L_pos3, nad4_pos1, nad6_pos1 | mtInv+F+I+I+R5 |
|  | P9 | nad5_pos1 | LG+F+I+G4 |
|  | P10 | rrnL, trnC, trnD, trnH, trnL1, trnS2 | Blosum62+F+R4 |
|  | P11 | rrnS, trnA, trnE, trnG, trnL2, trnQ, trnR, trnS1, trnT, trnV, trnW, trnY | Blosum62+F+R4 |
|  | P12 | trnF, trnN, trnP | Blosum62+F+G4 |
|  | P13 | trnI, trnK, trnM | Blosum62+F+I+G4 |
|  |  |  |  |
